# Consequences of interspecific plant hybridization on metabolic diversity in naturally occurring hybrid swarms

**DOI:** 10.1111/tpj.70444

**Published:** 2025-08-26

**Authors:** Olga Zafra‐Delgado, Fabian Schneider, Yoko Nakamura, Michael Reichelt, Jonathan Gershenzon, Frank H. Hellwig, Tobias G. Köllner

**Affiliations:** ^1^ Department of Biochemistry Max Planck Institute for Chemical Ecology Jena Germany; ^2^ Institute of Ecology and Evolution, Friedrich Schiller University Jena Germany; ^3^ Research Group Biosynthesis/NMR Max Planck Institute for Chemical Ecology Jena Germany; ^4^ Department of Natural Product Biosynthesis Max Planck Institute for Chemical Ecology Jena Germany

**Keywords:** plant interspecific hybridization, metabolic diversity, latitudinal gradient, geographical differentiation, hybrid swarm

## Abstract

Interspecific hybridization has influenced plant evolution and diversification. However, how hybridization may affect metabolic diversity, especially in naturally occurring hybridization zones, is unclear. In this study, we selected a *Baccharis* (Asteraceae) hybrid complex consisting of *B. linearis*, *B*. *macraei*, and *B. × intermedia* and characterized its metabolic profiles in multiple hybridization zones in central Chile to determine how hybridization affects plant chemistry. Untargeted liquid chromatography–time of flight mass spectrometry analysis of a total of 411 plant individuals collected in the field revealed that the hybrid *B. × intermedia* combines the metabolic profiles of its two parental species, *B. linearis* and *B*. *macraei*, independent of season, location, and environment. This combinatorial effect was observed in the specialized metabolites, while the primary metabolism did not differ between species. The metabolic diversity of the hybrid exceeded that of the parental species and was influenced by latitude, with higher metabolic diversity in the northern populations than in those in the south. In summary, our results demonstrate that natural interspecific hybridization can quickly increase the diversity of specialized metabolites. This could enhance protection against biotic or abiotic stressors, particularly in changing environmental conditions.

## Significance Statement

Studies on the effect of hybridization on plant chemistry have mainly been conducted with crop plants and artificially created hybrids, and the few studies that have analyzed naturally occurring hybrids have either examined only a small number of plants and/or only a small number of metabolites. We analyzed more than 400 field‐sampled individuals of a naturally occurring hybrid complex using (un)targeted metabolomics and NMR analysis to gain a comprehensive insight into its chemistry.

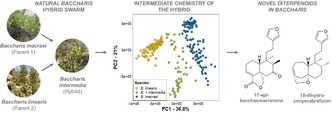

## INTRODUCTION

Natural hybridization has been recognized as a common phenomenon among vascular plants (López‐Caamal & Tovar‐Sánchez, 2014), associated with plant speciation (Soltis & Soltis, 2009), species merging, genetic swamping, and transfer across species (Seehausen, 2004). Since historical hybridization events account for 30%–70% of modern plant species, plant hybridization may have also played a role in the diversification of specialized plant metabolism (Kirk et al., 2005). Specialized metabolites play important ecological roles in defense against herbivores and pathogens, mediate plant–plant signaling, attract pollinators and herbivore predators, and confer protection against abiotic stressors (Moore et al., 2014). Therefore, plant hybridization may not only affect plant evolution but also its interaction with the environment (Cheng et al., 2011; Orians, 2000). Often, but not always, hybridization is followed by genome doubling. The resulting allopolyploid hybrids may exhibit some advantages over their diploid parents, phenotypically apparent as heterosis due to gene dosage effects or as changes in the reproductive system (Osabe et al., 2012; Qiu et al., 2020 for review). In general, metabolic diversity in first‐generation (F_1_) hybrids can be different from that of the parents because the hybrids could (a) produce all the specialized metabolites of both parents, (b) produce a selection of them, or (c) produce novel specialized metabolites (Cheng et al., 2011). At the level of large segregating populations (F_2_, F_3_, …, F_n_) with backcrossing to the parental species, greater chemical diversity is expected since all combinations of genes and alleles from both parental species may be present (Cheng et al., 2011; Kirk et al., 2005). Furthermore, plant hybridization can also affect the quantitative levels of specialized metabolites in the hybrid, as they can produce similar, higher, intermediate, or lower levels than the parental species (Cheng et al., 2011). Natural hybridization zones, which represent a true window into how hybridization affects plant evolution, can be useful to determine the effects of hybridization on the diversity of plant specialized metabolites. However, little attention has been paid to this area of research, probably because the parent species and their hybrids are often difficult to distinguish in the field.

Interspecific hybridization is considered a widespread phenomenon in the genus *Baccharis* L. (Asteraceae), although little attention has been paid to its role in shaping the diversity, distribution, evolution, and ecology of this genus (Heiden, 2021). With about 440 species distributed throughout the Americas, *Baccharis* is one of the largest genera in the Asteraceae, mainly comprising evergreen, dioecious, insect‐pollinated shrubs (Heiden, 2021). In Chile, previous morphological studies have resulted in the identification of only 16 species, but 27 interspecific hybrids, highlighting the important role of plant hybridization in shaping *Baccharis* spp. populations (Faini et al., 1991; Hellwig, 1990). However, in contrast to many other hybrid complexes, hybrid formation in *Baccharis* is not followed by polyploidization. All Chilean *Baccharis* species for which chromosome counts have been performed were found to be diploid (Hellwig, 1990; Schneider, 2025). Among the different *Baccharis* spp. complexes, the easily detectable hybrid *B. × intermedia*, which results from the interspecific hybridization of *B. macraei* and *B. linearis*, has already been described in more detail (Faini et al., 1991; Schneider & Hellwig, 2024). While *B. macraei* grows exclusively on sandy soils close to the seashore in central Chile, *B. linearis* is one of the most widespread and abundant taxa of *Baccharis* in Chile, ranging from the coast to the mountains and covering mainly areas where the primary vegetation has been disturbed. *B. × intermedia* has been described to frequently occur in the contact zone of the two parental species. Although the hybrid origin of *B. × intermedia* has been demonstrated by morphological and phytochemical studies (Faini et al., 1991) and SNP characterization (Schneider & Hellwig, 2024), little is known about the consequences of hybridization in shaping the diversity of specialized metabolites in this species. Recent work suggests that the metabolic profile of *B. × intermedia* is the combination of the two parental chemistries; however, chemical characterization was limited to only a few specialized metabolites and to plants from a single location (Faini et al., 1991).

In this study, we used untargeted liquid chromatography–time of flight mass spectrometry (LC‐qTOF‐MS) to characterize the metabolomes of *B. × intermedia, B. macraei*, and *B. linearis* in depth by examining several naturally occurring hybridization zones located within a north–south range of more than 400 km in central Chile. Specifically, we sampled plants from hybrid zones and reference populations of parental species in multiple locations along the coastal area and at two different seasons to characterize the metabolic diversity of this *Baccharis* species complex across locations, environmental conditions, and seasons. To further identify the effect of hybridization on the chemistry of subsequent advanced generations (F_2_, F_3_, …, F_n_), we grew and analyzed plants from seeds collected from individuals morphologically characterized as *B. × intermedia, B. macraei*, and *B. linearis*.

## RESULTS

### The hybrid *B. × intermedia* has an intermediate metabolic profile compared with its parents, *B. linearis* and *B. macraei*

**Figure 1:**
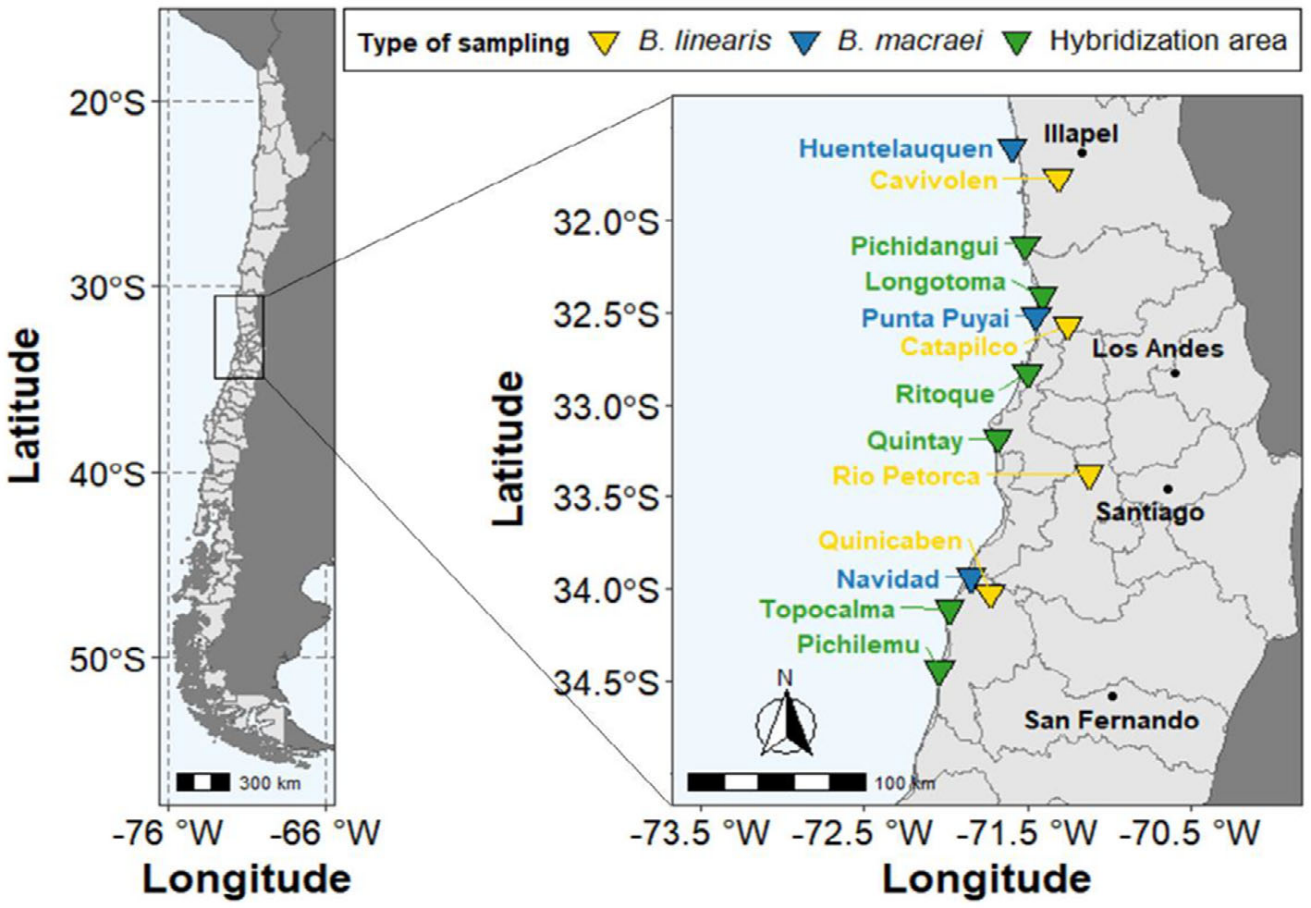
Sampling of *Baccharis* hybrid swarms and reference parental populations in central Chile. Map shows the location of the different field collection sites, distributed across the coastal areas of Chile. Yellow triangles represent *B. linearis* reference populations; blue triangles represent *B. macraei* reference populations; green triangles represent hybridization zones (transects).

Plants were collected along different transects extending from sandy beach soils inland, where morphological evidence of interspecific hybridization was found. For comparison purposes, *B. linearis* reference populations were found inland at different latitudes, whereas *B. macraei* reference populations were located in coastal areas with no evidence of hybridization and no surrounding *B. linearis* populations. The location of the different transects (hybridization zones) and reference populations is visualized in Figure 1. A total of 411 plant individuals were sampled and analyzed.

**Figure 2:**
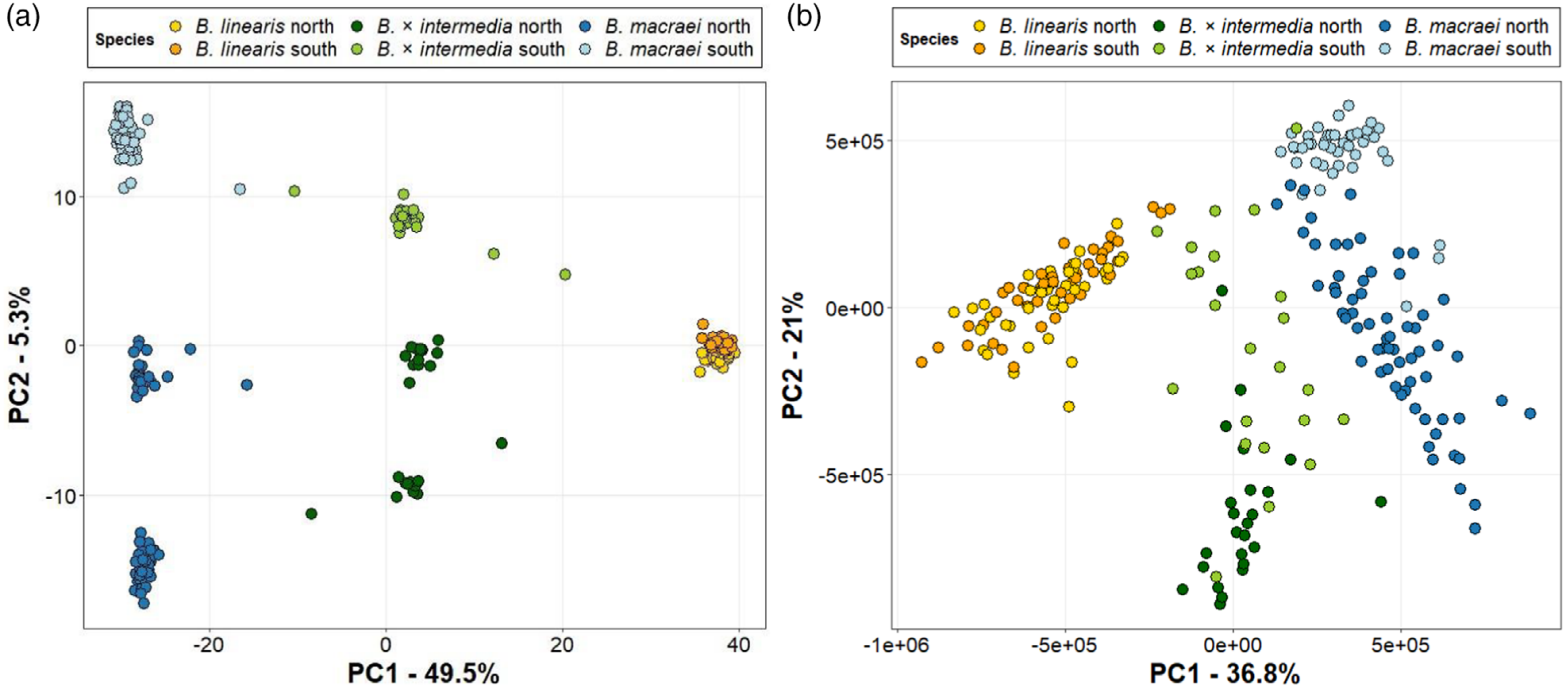
Principal component analysis based on (a) Genotype‐by‐sequencing (GBS) data with genetic clustering (*K* = 6) and (b) profiles of semi‐polar metabolites obtained by LC‐MS measurements of methanolic leaf extracts, for *B. macraei*, *B. linearis*, and *B. × intermedia*. Samples in (b) are colored according to the GBS‐based species identification shown in (a). Yellow circles, *B. linearis* north; orange circles, *B. linearis* south; dark blue circles, *B. macraei* north; light blue circles, *B. macraei* south; dark green circles, *B. × intermedia* north, and light green circles, *B. × intermedia* south.

Methanol extracts from leaves collected at the different sampling locations were analyzed by LC‐qTOF‐MS, and results were visualized with a PCA, colored according to a preliminary morphological identification made in the field (Figure S2). This PCA revealed three different metabolic profiles, mostly corresponding to individuals preliminarily identified as *B. linearis*, *B. × intermedia*, or *B. macraei*. However, a number of ambiguities could be found, especially in those plants originally classified as hybrids (Figure S2). Thus, to increase the confidence on the species identification, samples from four transects (Pichidangui, Pichilemu, Quintay, Topocalma) and from two reference populations of *B. linearis* and *B. macraei* were subjected to genetic analysis (GBS). In fact, most of the genetically analyzed plants were found to have been assigned correctly by the prior morphological identification, although a number of individuals morphologically identified as *B. × intermedia* belonged genetically to *B. macraei* or *B. linearis* (Figure S3A). In a PCA based on the GBS data, the different *Baccharis* species were well separated and formed distinct groups, including the hybrid *B. × intermedia*, which grouped midway between *B. macraei* and *B. linearis* (Figure 2a). There is evidence of limited backcrossing between the hybrid and the parental species, as there were only four individuals intermediate to *B. × intermedia* and *B. macraei* and three between *B. × intermedia* and *B. linearis*. While *B. linearis* formed a densely packed cluster, *B. macraei* and *B. × intermedia* each formed three distinct groups, which correspond to specific geographic areas, respectively. While the northern subgroup of *B. macraei* included samples from Pichidangui and Huentelauquén, the southern subgroup included samples from Navidad, Pichilemu, and Topocalma (Figure S3B). Between these two subgroups, we found the Quintay individuals, which were distinct from the other two subgroups but clearly belonged to the northern *B. macraei* cluster (Figure 2a; Figure S3B). Similarly, the hybrid *B. × intermedia* was divided into three subgroups, following the same pattern as *B. macraei*.

To determine whether the GBS‐based species identification correlated with differences in metabolic profiles, we performed a PCA on the untargeted metabolomics data (2049 features). Similarly to the PCA based on the GBS data, *B. macraei*, *B. × intermedia*, and *B*. *linearis* formed distinct clusters (Figure 2b). Since *B. × intermedia* grouped in between the two parental species, the hybrid chemistry seems to be a combination of *B. macraei* and *B. linearis*. While there were no clear differences between the metabolic profiles of the northern and southern populations of *B. linearis*, both the northern and southern populations of *B. macraei* and *B. × intermedia* differed in their metabolomes and showed distinct chemotypes (Figure 2b).

To prove the intermediate nature of the hybrid's chemistry, we extracted the 20 features that contributed most to the separation in the first principal component and compared their relative abundances in the different species. As observed in Table 1, the hybrid plants contained intermediate levels for 18 out of the 20 features, suggesting that the chemistry of the hybrid is indeed the result of the combination of the parental metabolic profiles. Moreover, the construction of molecular networks by clustering the mass spectra according to similarity and coloring each node according to the abundance of the corresponding metabolite in each species revealed that specific features from each parental species were also found at variable levels in the hybrids (Figure S4–S7).

**Table 1:** Top 20 features contributing the most to the separation of chemotypes in the PCA shown in Figure 2b, colored according to the relative quantitation level (yellow (high), green (intermediate), blue (low)).

Feature ID	Exact mass	Retention time (min)	Molecular formula	Putative annotation	*B. linearis*	*B. × intermedia*	*B. macraei*	Type of ion
171	284.2140	1.77	C20H28O		39643±8977 (a)	2462±1626 (b)	233±27 (b)	[M + H]^+^
196	304.2039	1.89	C19H28O3	steroid lacton	36481±1661 (a)	1685±263 (b)	139±25 (b)	[M + H]^+^
198	334.2144	1.89	C20H30O4	diterpenoid	113594±5234 (a)	5008±826 (b)	429±83 (b)	[M + H]^+^
212	298.1933	1.89	C20H26O2	retinoid	58062±2647 (a)	2745±432 (b)	312±44 (b)	[M + H]^+^
385	272.2140	2.98	C19H28O	17‐hydroxysteroid	34522±2768 (a)	1940±271 (b)	195±19 (b)	[M + H]^+^
1217	498.1162	1.22	C25H22O11	N‐acyl‐alpha amino acid or derivatives	122698±6011 (a)	76365±6029 (b)	57316±5038 (b)	[M + H]^+^
1265	246.0892	1.66	C14H14O4	naphthalen	46777±1641 (a)	20107±1539 (b)	393±30 (c)	[M + H]^+^
1281	302.2245	1.77	C20H30O2	diterpenoid	36426±8405 (a)	2273±1477 (b)	242±25 (b)	[M + H]^+^
1294	352.2249	1.89	C20H32O5	kaurane diterpenoid	194409±10433 (a)	5366±967 (b)	373±81 (b)	[M + H]+
954	316.2038	1.89	C20H28O3	steroid or derivative	46193±2056 (a)	2270±351 (b)	297±35 (b)	[M + H]+
1324	286.1933	1.89	C19H26O2	estrogen or derivative	95056±4346 (a)	4282±704 (b)	362±64 (b)	[M + H]^+^
1384	322.2508	2.36	C20H34O3	diterpenoid	100±30 (a)	22586±2399 (b)	60223±6279 (c)	[M + H]+
1428	230.0943	2.55	C14H14O3	aryl ketones	43563±1638 (a)	13111±1038 (b)	476±29 (c)	[M + H]+
1430	316.0582	2.60	C16H12O7	3‐*O*‐methylated flavonoid	46485±2552 (a)	118312±10710 (b)	90748±7755 (c)	[M + H]^+^
1472	316.0582	2.88	C16H12O7	3‐*O*‐methylated flavonoid	318366±12451 (a)	180622±13454 (b)	23753±2256 (c)	[M + H]^+^
1545	300.0633	3.44	C16H12O6	*O*‐methylated flavonoid	15713±1390 (a)	129429±16015 (b)	341467±24089 (c)	[M + H]^+^
1605	300.0634	3.84	C16H12O6	*O*‐methylated flavonoid	126080±11044 (a)	175653±22731 (ab)	169696±11516 (b)	[M + H]^+^
1628	330.0740	3.93	C17H14O7	3′‐*O*‐methylated flavonoid	97358±6246 (b)	37510±3668 (a)	30500±2211 (a)	[M + H]^+^
1667	150.1045	4.11	C10H14O		108525±8631 (b)	6187±856 (a)	518±71 (a)	[2M+H]^+^
1668	336.2300	4.11	C18H30N3O3	colensane or clerodane diterpenoid	46564±3732 (b)	2351±342 (a)	405±32 (a)	[M+Na]^+^

*Notes*: Values represent the mean peak height ± standard error for each group (*B. linearis*, *n* = 86; *B. × intermedia*, *n* = 47; *B. macraei*, *n* = 109). Different letters denote statistically significant differences among the means according to ANOVA (Tukey test) analysis (*P* < 0.05). Features are shown in descending order. Please note that because in‐source fragmentation was not considered in the bucketing process, features with the same retention time may represent different in‐source fragments originating from the same compound.

### Intermediate chemistry of hybrids is due to differences in specialized, not primary metabolites

**Figure 3:**
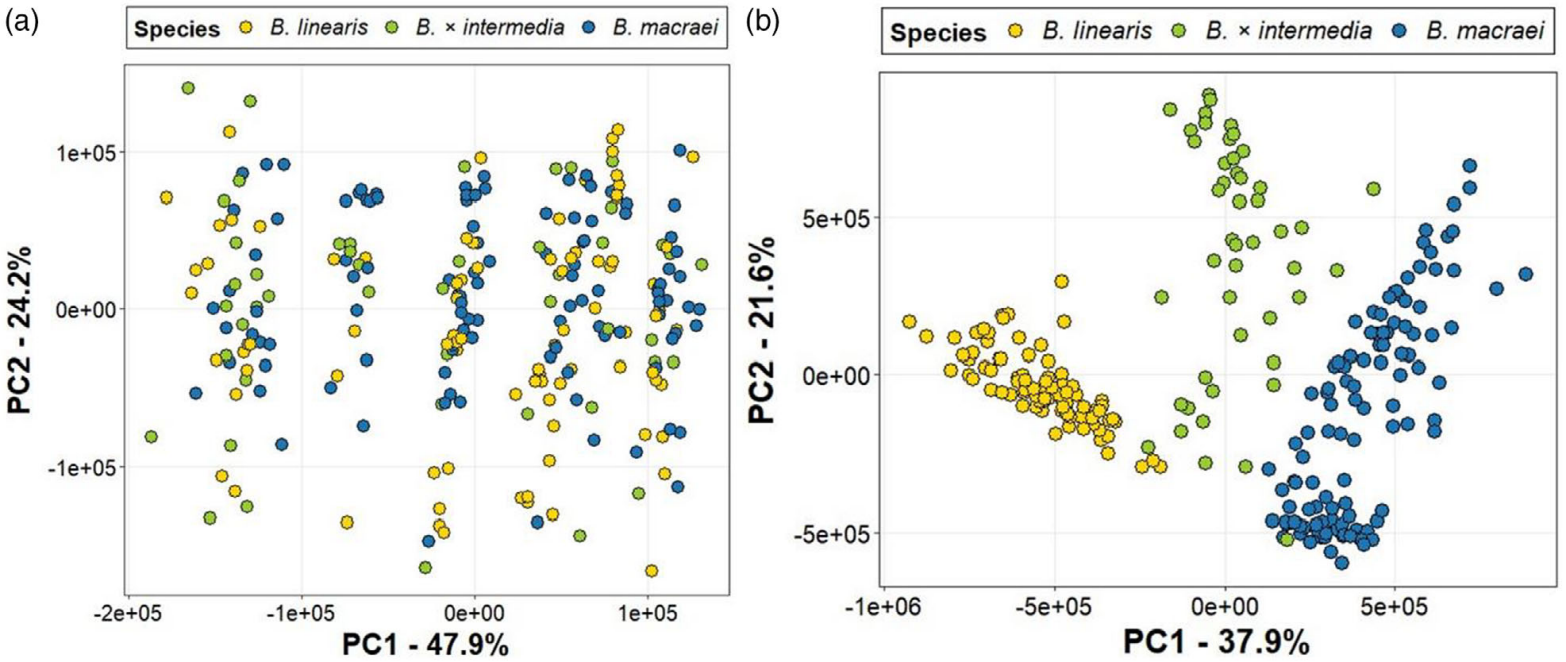
Principal component analysis based on the profiles of the metabolic features annotated as (a) primary metabolites and (b) specialized metabolites, obtained by LC‐MS measurements of methanolic leaf extracts from *B. macraei*, *B. linearis*, and *B. × intermedia*. Yellow circles, *B. linearis*; blue circles, *B. macraei*; green circles, *B. × intermedia*.

Since almost all of the 20 features that contributed most to the species‐specific separation in the PCA shown in Figure 2b were annotated as specialized metabolites (Table 1), we wondered whether this separation was, in general, caused by differences in specialized metabolism. To address this question, the metabolic features were sorted into primary (342 features) and specialized (1707 features) metabolites based on the prediction of their chemical class (Figure S8), and the two subsets were subjected to PCA separately. While the species‐specific separation observed in the PCA with all features (Figure 2b) was maintained when PCA was performed on the subset of specialized metabolites, it was completely lost when only primary metabolites were included in the PCA (Figure 3). Notably, the PCA based on primary metabolites revealed five distinct clusters (Figure 3a), which we hypothesized might represent different sampling locations. However, coloring of individual plants in this PCA according to their respective sampling locations did not reveal any clustering (Figure S9), suggesting that factors other than location were responsible for this grouping.

### Neo‐clerodane diterpenoids contribute to the intermediate chemistry of the hybrids

**Figure 4:**
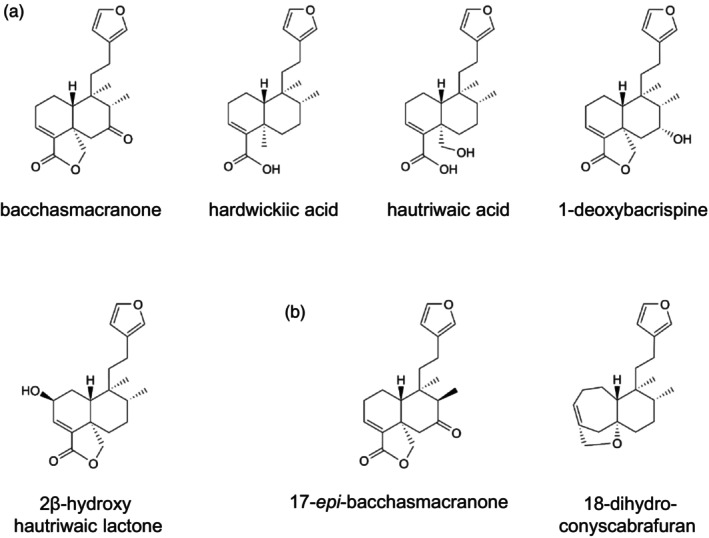
Known (a) and novel (b) neo‐clerodane diterpenoids isolated from *Baccharis macraei*. Compounds were isolated from leaves and structures were elucidated using MS and NMR analysis. While five of the isolated neo‐clerodane diterpenoids have already been reported in the literature, two were novel.

Although little is known about the chemistry of the hybrid complex of *B. macraei, B*. *linearis*, and *B. × intermedia*, differences in the diterpenoid profiles of the two parental species have been reported in the literature, particularly in the neo‐clerodane diterpenoid pathway (Gambaro et al., 1986; He et al., 1996). To further explore these differences and to investigate the influence of hybridization on characteristic diterpenoids, we selected metabolic features for isolation and identification that were annotated as diterpenoids in the metabolomes and were abundant in at least one of the *Baccharis* species studied for isolation and identification. Mass spectrometry and NMR analyses revealed that four of the six compounds isolated had previously been described as neo‐clerodane diterpenoids in *Baccharis*, including hautriwaic acid, 2β‐hydroxy‐hautriwaic lactone, 1‐deoxybacrispine, and bacchasmacranone, while the remaining two compounds, also identified as neo‐clerodane diterpenoids and named 8‐*epi*‐bacchasmacranone and 18‐dihydro‐conyscabrafuran, were novel natural compounds (Figure 4). Notably, hardwikiic acid, another neo‐clerodane diterpenoid, could also be identified in the metabolome by comparing its retention time and MS–MS spectrum with those of a commercially available standard. As shown in Figure S10, the identified diterpenoids showed either intermediate (bacchasmacranone, 8‐*epi*‐bacchasmacranone), similar (2β‐hydroxy‐hautriwaic lactone, hautriwaic acid, 18‐dihydro‐conyscabrafuran, 1‐deoxybacrispine), or even higher (hardwikiic acid) accumulation in the hybrids compared with the parental species.

### Plant sex and season had nearly no effect on the chemical differences among species

Since the genus *Baccharis* is dioecious, we investigated whether the sex of the plants influenced the chemical profiles. As shown in Figure S11, no clustering between the chemical profiles of male and female plants was observed in any of the three species examined, indicating that plant sex does not affect chemistry. To determine whether season has an effect on the chemical differentiation between species, we compared the metabolomes of a subset of plants (*n* = 160) from different sites (Pichidangui, Quintay, Cavivolén, Navidad, Punta Puyai, Pichilemu, and Catapilco) that were first sampled in the fall (March 2022) and again the following spring (September 2022). The general clustering between the different species was maintained in the spring and fall samples, although the differences between *B. macraei, B. linearis*, and *B. × intermedia* were slightly smaller in the spring samples (Fig. S11), likely due to plant dormancy.

### Metabolite diversity is higher in the hybrids than in the parental species

**Figure 5:**
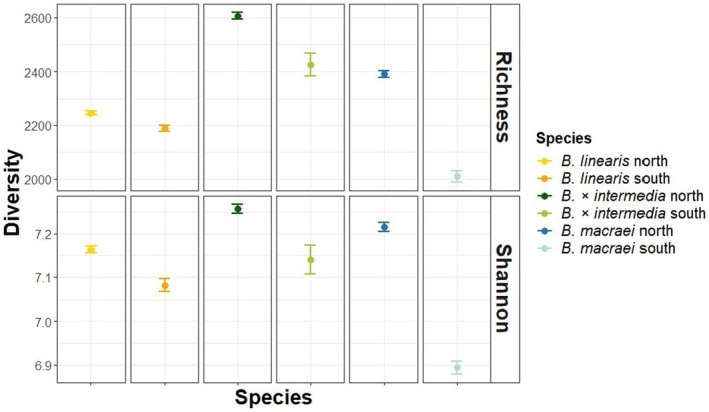
Diversity indexes of metabolites based on richness and Shannon indices for the different species and subpopulations.

To investigate whether *B. × intermedia* has a greater diversity of metabolites compared with the parental species, we calculated two metrics of metabolite diversity: (a) the number of metabolites within an individual plant (metabolite richness) and (b) the abundance‐weighted diversity of metabolites, expressed as the Shannon–Weaver index based on metabolite intensities at the plant individual level (Shannon, 1948). As shown in Figure 5, the northern populations of *B. macraei* and *B. × intermedia* each had higher metabolite richness than their respective southern populations. In addition, the hybrid *B. × intermedia* generally showed higher richness than the two parental species *B. macraei* and *B. linearis*. Shannon diversity also showed that *B. × intermedia* (north and south) had the highest diversity compared with the respective parental species.

### The intermediate phytochemistry of the hybrid is retained in its offspring in a greenhouse experiment

**Figure 6:**
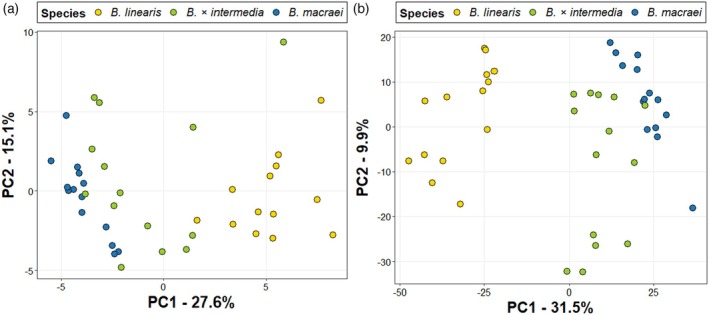
PCA based on (a) volatile (hexane‐soluble) and (b) non‐volatile (methanol‐soluble) leaf metabolites of offspring individuals of *B. macraei*, *B. linearis*, and *B. × intermedia* grown under controlled greenhouse conditions. Leaves from 1‐year‐old plants were harvested and extracted with hexane (a) and methanol (b). Hexane extracts were analyzed using GC‐MS while methanol extracts were analyzed using LC‐qTOF in positive mode. Species assignment was done based on the morphological identification of the parents in the field (Schneider & Hellwig, 2024). Yellow circles, *B. linearis*; green circles, *B. × intermedia*; blue circles, *B. macraei*.

To generate plants of *B. macraei*, *B. × intermedia*, and *B*. *linearis* for chemical characterization under controlled greenhouse conditions, we grew individuals from field‐collected seeds of plants morphologically characterized as *B. macraei*, *B. × intermedia*, and *B*. *linearis* in a greenhouse at the Botanical Garden Jena, Germany. As shown in Figure 6, PCA of the volatile profiles and the non‐volatile metabolomes revealed that *B. × intermedia* clustered separately from *B. macraei* and *B. linearis* and in an intermediate position, suggesting that the progeny showed an intermediate chemistry as we observed in the populations sampled in the field in 2022. However, for volatiles as well as for non‐volatile compounds, *B. × intermedia* clustered closer to *B. macraei* than to *B. linearis* (Figure 6), suggesting that the *B. × intermedia* offspring plants might be backcrosses to *B. macraei*, rather than *B. linearis*. A detailed analysis of volatile leaf metabolites showed that the profiles of all three species were dominated by mono‐, sesqui‐, and diterpenoids (Table 2). While *B. linearis* contained mainly mono‐ and sesquiterpenoids, *B. macraei* accumulated predominantly diterpenoids. In contrast, *B. × intermedia* combined the chemistry of the parental species and produced all types of terpenoids in intermediate or even highest amounts (Table 2).

**Table 2:** *B. macraei, B. × intermedia*, and *B. linearis* differ in their volatile profiles.

Tentative identification	*B. linearis*	*B. × intermedia*	*B. macraei*
Green leaves volatiles			
2‐hexenal	20.6 ± 4.0 (a)	28.9 ± 7.7 (a)	29.3 ± 6.3 (a)
nonanal* (KI:1104)	4.5 ± 0.5 (a)	3.5 ± 0.4 (ab)	2.8 ± 0.1 (b)
Monoterpenes			
α‐pinene** (KI:927)	96.7 ± 16.0 (a)	38 ± 9.8 (b)	10.9 ± 2.9 (b)
β‐pinene** (KI:973)	516.1 ± 57.7 (a)	132.2 ± 19.1 (b)	23.2 ± 3.5 (b)
β‐myrcene* (KI:991)	44.7 ± 5.9 (a)	15.4 ± 3.9 (b)	5.0 ± 1.1 (b)
d‐limonene** (KI:1030)	448.4 ± 50.8 (a)	101.7 ± 27 (b)	21.0 ± 5.2 (b)
β‐ocimene* (KI:1049)	5.4 ± 0.6 (a)	4.2 ± 1 (a)	0.5 ± 0.1 (b)
γ‐terpinene (KI:1059)	2.6 ± 0.8 (a)	0.4 ± 0.1 (b)	0.2 ± 0.1 (b)
terpinolene* (KI:1089)	3.3 ± 0.7 (a)	1.2 ± 0.3 (ab)	0.5 ± 0.1 (b)
Sesquiterpenes			
γ‐elemene (KI:1311)	12.3 ± 0.9 (a)	2.2 ± 0.4 (b)	0.9 ± 0.1 (b)
α‐cubebene** (KI:1354)	3.1 ± 0.3 (a)	2.0 ± 0.5 (ab)	1.1 ± 0.1 (b)
cyclosativene* (KI:1371)	14.3 ± 1.6 (a)	8.1 ± 1.5 (b)	3.8 ± 0.3 (c)
α‐copaene** (KI:1380)	47.2 ± 7.2 (a)	24.2 ± 5.6 (b)	12.8 ± 1.3 (b)
methyl eugenol* (KI:1405)	3 ± 0.7 (a)	7.5 ± 1.8 (b)	3.8 ± 0.6 (ab)
β‐caryophyllene (KI:1425)	101.2 ± 26.4 (a)	40 ± 8.9 (b)	7.5 ± 0.7 (b)
β‐copaene* (KI:1434)	4.7 ± 0.5 (a)	1.1 ± 0.3 (b)	0.2 ± 0.1 (b)
α‐humulene** (KI:1460)	24.2 ± 1.7 (a)	54.9 ± 11.3 (b)	21.8 ± 4.7 (a)
γ‐murolene** (KI:1471)	3.1 ± 0.2 (a)	23.1 ± 14.9 (a)	1.2 ± 0.2 (a)
germacrene D* (KI:1488)	255.6 ± 25.2 (a)	75.1 ± 19.2 (b)	31.1 ± 4.4 (b)
bicyclogermacrene* (KI:1494)	78.2 ± 37.3 (a)	63.2 ± 16.1 (a)	14.7 ± 4.2 (a)
germacrene B* (KI:1530)	5.0 ± 3.0 (a)	3.4 ± 1.1 (a)	1.1 ± 0.3 (a)
globulol (KI:1611)	4.8 ± 0.6 (a)	7.0 ± 1.8 (a)	3.5 ± 0.4 (a)
*epi*‐cubebol* (KI:1628)	10.4 ± 1.1 (a)	1.2 ± 0.3 (b)	0.4 ± 0.1 (b)
γ‐cadinol (KI:1653)	2.3 ± 0.3 (a)	1.6 ± 0.4 (ab)	0.9 ± 0.4 (b)
germacrene‐d‐ol/spathulenol (KI:1583)	19.0 ± 2.3 (ab)	22.2 ± 4.9 (a)	9.8 ± 0.8 (b)
isoaromadendrene epoxide (KI:1592)	5.1 ± 1.2 (a)	5.1 ± 1.7 (a)	1.6 ± 0.3 (a)
ent‐germacra‐4(15),5,10(14)‐trien‐1‐ol (KI:1696)	6.0 ± 1.3 (a)	11.3 ± 2.1 (b)	2.6 ± 0.6 (a)
shyobunol (KI:1703)	7.0 ± 1.5 (a)	106.1 ± 25.7 (b)	44.9 ± 5.8 (a)
Fatty acids			
methyl (*Z*)‐dec‐2‐en‐4,6‐diynoate (KI:1503)	306.0 ± 38.2 (a)	43.0 ± 7.0 (b)	20.8 ± 3.6 (b)
methyl (*Z*)‐dec‐2‐en‐4,6‐diynoate (KI:1511)	2607.0 ± 199.6 (a)	43.3 ± 19.6 (b)	6.2 ± 0.4 (b)
1,8,11‐heptadecatriene (KI:1663)	1.9 ± 0.5 (a)	8.8 ± 3.2 (b)	2.1 ± 0.3 (a)
1,8,11,14‐heptadecatetraene (KI:1669)	19.0 ± 16.5 (a)	4.9 ± 1.9 (a)	0.8 ± 0.2 (a)
Diterpenes			
neophytadiene (KI:1838)	10.6 ± 0.8 (a)	7.7 ± 0.9 (b)	6.9 ± 0.7 (b)
bacchasmacranone**	3.4 ± 0.7 (a)	31.7 ± 7.9 (b)	59.5 ± 11.1 (c)
unidentified diterpene 1	1.6 ± 0.4 (a)	24.4 ± 12.2 (a)	0.1 ± 0.1 (a)
unidentified diterpene 2	0.9 ± 0.2 (a)	1.4 ± 0.4 (a)	1 ± 0.3 (a)
unidentified diterpene 3	1.1 ± 0.4 (a)	0.3 ± 0.1 (a)	0.3 ± 0.1 (a)
unidentified diterpene 4	9.1 ± 2.8 (a)	2.7 ± 1.3 (a)	6.9 ± 2.4 (a)
unidentified diterpene 5	1.4 ± 0.3 (a)	3.6 ± 1.1 (ab)	6.1 ± 1 (b)
Unidentified diterpene 6	6.9 ± 1.6 (a)	11.2 ± 3.5 (a)	11.7 ± 3.3 (a)
unidentified diterpene 7	3 ± 0.7 (a)	10.4 ± 2.7 (ab)	18.9 ± 3.6 (b)
Unidentified			
unidentified 1 (KI:1444)	10.4 ± 0.8 (a)	4.1 ± 0.7 (b)	1.1 ± 0.2 (c)
unidentified 2 (KI:1572)	29.9 ± 4.0 (a)	1.6 ± 0.4 (b)	0.5 ± 0.1 (b)
unidentified 3	5.3 ± 4.3 (a)	0.7 ± 0.3 (a)	0.1 ± 0.1 (a)

*Notes*: Volatile compounds were extracted with hexane from leaf powder and qualitatively and quantitatively analyzed by GC‐MS and GC‐FID, respectively. The abundance of volatile compounds is given in ng per mg fresh weight and the average and SE (*n* = 3) are shown. For better visualization, relative concentrations in the different species are colored according to the average level: yellow (high), green (intermediate), blue (low). Volatiles identified using authentic standards are marked with **, while volatiles tentatively identified using Kovats' indices (KI) are marked with *. Different letters denote statistically significant differences among the means according to ANOVA (Tukey test) analysis (*P* < 0.05).

## DISCUSSION

In this work, we studied a naturally occurring *Baccharis* hybrid complex, consisting of the two parental species *B. macraei* and *B. linearis*, and the hybrid *B. × intermedia*, to understand how natural hybridization can affect plant chemistry.

Prior to this study, little was known about the chemistry of the parental species, apart from some differences reported for the specialized metabolite profiles. For instance, Faini et al. (1991) reported that *B. linearis* is mainly characterized by the presence of lachnophyllum ester, the aromatic compound chromene, the sesquiterpene spathulenol, and three 3‐methoxyflavonoids, while *B. macraei* produces four neo‐clerodane diterpenoids and two 6‐methoxyflavonoids as characteristic components. Our untargeted metabolomics study underscored the metabolic differences between *B. macraei* and *B. linearis* and revealed clusters of chemically related specialized metabolites characteristic for one of the two species (Figure S4–S7). These metabolite clusters, preliminarily annotated as diterpenoids, triterpenoids, or flavonoids, highlight specific metabolic pathways that contribute to the chemical distinctiveness of *B. macraei* and *B. linearis*. We could further confirm the presence of several neo‐clerodane diterpenoids by mass spectrometry and NMR analysis, including hardwickiic acid, hautriwaic acid, 2β‐hydroxy‐hautriwaic acid, bacchasmacranone, and 1‐deoxybacrispine, and two novel compounds 8‐*epi*‐bacchasmacranone and 18‐dihydro‐conyscabrafuran, which were exclusive to or at least more abundant in *B. macraei* compared with *B. linearis* (Figure S10).

The aforementioned study by Faini et al. (1991) showed that the hybrid *B. × intermedia* produced all of the specialized compounds of the parental species, suggesting an additive chemotype. However, because the number of compounds analyzed by thin‐layer chromatography in this study was very small, this statement was limited. The positioning of *B. × intermedia* between the two parental species in our metabolome‐based PCA (Figure 2b) strongly supports the additive chemistry reported by Faini and coworkers. The visualization of molecular networks (Figure S4–S7) and the relative quantification of the 20 features that contributed most to the PCA separation (Table 1) showed that the chemistry of *B. × intermedia* is overall the result of a combination of the two parental chemistries. This is similar to previous hybridization studies on other wild plants, which showed that if both parents or only one of them produce a compound, hybrids often produce it as well (Cheng et al., 2011; Orians, 2000). However, to the best of our knowledge, this is the most comprehensive study performed so far, which highlights the additive or combinatorial nature of hybrid chemistry, as previous studies focused on a reduced number of compounds or only on compounds detectable by NMR, a far less sensitive technique (Cheng et al., 2011; Kirk et al., 2005; Orians, 2000).

Specialized metabolites produced by plants are mainly used for defense or communication. Primary metabolites, on the other hand, are essential for basic life processes. This is reflected in the fact that specialized metabolism tends to be highly variable between species, while primary metabolism is highly conserved. Indeed, our results showed that *B. macraei*, *B. linearis*, and *B. × intermedia* significantly differed in their specialized, but not in their primary metabolites; that specialized metabolites mainly contribute to the combinatorial chemistry of hybrids (Figure 3). Moreover, the terpene profiles of plants grown under controlled greenhouse conditions showed also a combinatorial chemistry, suggesting that the metabolic consequences of interspecific hybridization in *Baccharis* persist in later generations.

Sexual dimorphism affecting leaf metabolites has been documented in certain plant genera (Bañuelos & Obeso, 2004; Rabska et al., 2020). However, we found no evidence of sexual dimorphism in the leaf metabolomes of *B. linearis* and *B. macraei*, which is consistent with analogous studies in other *Baccharis* species, such as *B. salicifolia*, *B. articulata*, and *B. tridentata*, where no significant differences in volatile profiles were observed between male and female plants (Minteguiaga et al., 2015; Minteguiaga et al., 2021; Tomé et al., 2019).

In principle, hybridization can lead to a restructuring of metabolic pathways by allele complementation or genetic rearrangement, which in turn could result in the production of new metabolites or the accumulation of pathway intermediates in the hybrids (Cheng et al., 2011; Orians, 2000). The increased metabolic richness and Shannon diversity index of *B. × intermedia* compared with *B. macraei* and *B. linearis* indeed suggest the presence of novel metabolites in the hybrid (Figure 5). However, more targeted chemical approaches and metabolite characterization are required to further substantiate this hypothesis. It is noteworthy that both *B. macraei* and *B. × intermedia* populations in the north showed higher metabolic richness and Shannon diversity index than the populations in the south, which could be explained by the genetic diversity of *B. macraei* decreasing from north to south and a polytopic origin of hybrids that reflect this geographical differentiation, as already suggested by Schneider and Hellwig (2024).

While about a quarter of plant species appear to be involved in interspecific hybridization (Ellstrand et al., 1996; Mallet, 2005), the rate of hybrid speciation is much lower for various reasons (Paun et al., 2009). In principle, hybridization can result in homoploid or allopolyploid hybrid speciation. Homoploid hybrid speciation leads to a hybrid species that possesses the same number of chromosomes as the parental species, while allopolyploid hybrid speciation involves polyploidization and results in the formation of an allopolyploid species. Allopolyploidy is favored by a higher genomic divergence of the parental species and is generally associated with rapid reproductive isolation, which consequently results in the formation of a stable hybrid species (Paun et al., 2009). Homoploid hybrid speciation, however, is frequently observed when the parental species exhibit highly similar genome structures and appears to be favored by the availability of a suitable ecological niche or fitness peak and rapid chromosomal evolution (Buerkle et al., 2000; Mallet, 2007; Schumer et al., 2014). Since homoploid hybrids can also backcross with their parent species, complex hybrid swarms with different intermediate genotypes can be formed. In the *Baccaris* hybrid complex investigated in this study, the weak chromosomal differentiation between the parental species, probably due to dioecy, combined with the high fertility of the hybrids, prevents the development of allopolyploids. Chromosome counts have demonstrated that *B. macraei* and *B. linearis*, as well as their hybrids classified as *B. × intermedia*, are indeed diploid (Hellwig, 1990; Schneider, 2025). Thus, *B. × intermedia* could in principle produce offspring among sibling individuals or with a plant belonging to one of their parental species, while selfing is not possible due to dioecy. However, a very recent study by Schneider et al. (2025) revealed that most of the *B. × intermedia* plants examined are F1 hybrids and have only recently emerged. No plants resulting from the crossing of two F1 hybrids could be detected (Schneider et al., 2025). This could be due to the fact that the number of *B. × intermedia* plants in the field is small compared with the number of individuals of the parent species. Most of the female hybrid plants would therefore use pollen from the parent species for reproduction, making crossbreeding of two F1 hybrids rather unlikely. However, it cannot be ruled out that there are also intrinsic barriers to crossbreeding between *B. × intermedia* individuals. Future studies should therefore include controlled crossing experiments.

In summary, our results suggest that new F1 hybrids (*B. × intermedia*) are produced repeatedly in different locations. Since *B. × intermedia* plants are diploid as their parents and often grow in proximity to large populations of the parent species, backcrossing is favored over hybrid reproduction, making rapid hybrid speciation rather unlikely. However, the repeated formation of F1 hybrids with a combinatorial chemistry of specialized metabolites, as demonstrated in this study, creates a small but persistent pool of plants that exhibit greater chemical diversity than their parents. Because metabolic diversity is considered a driving force of adaptation (Petrén et al., 2024; Weng et al., 2021), these plants could adapt more quickly than their parent species to a changing environment. They could also occupy new ecological niches, which could enable the persistence of hybrids and ultimately lead to a stable hybrid species. In addition, the continuous gene flow between the parent species via the hybrids and their backcrosses has the potential to expand the chemical repertoire of the parent species through introgression.

## MATERIALS AND METHODS

### Plant collection

While *B. linearis* is a widespread shrub in inland areas in Chile, *B. macraei* grows exclusively on sandy coastal soils, ranging from latitude 31°S to 35°S (Faini et al., 1991). Between these two spatially largely separated ranges, the hybrid *B. × intermedia* is found at several localities where the parental species are in close contact. During the first field trip in March 2022, plants morphologically identified as *B. linearis*, *B*. *macraei*, or *B. × intermedia* were labeled and sampled along six longitudinal transects that always started at the coast and extended to a point between 50 m and 6 km inland. Fifty plant individuals were sampled per transect. For this purpose, twigs were collected at four different points in the canopy of a single plant, pooled into one sample, and stored in silica gel for 2 days at room temperature for both metabolomic and genomic analysis. In addition, morphological parameters, flowering stage, plant size, sex, and coordinates were recorded for every sampled plant. When plants were flowering, flowers were manually removed from the twigs to avoid extracting the metabolites from flowers. For each individual, voucher specimens were collected and deposited in the Herbarium Haussknecht (JE) (University of Jena, Germany). The different transects sampled (ordered from north to south) were: Pichidangui (32°07′ S, 71°30′ W), Longotoma (32°24′ S, 71°21′ W), Ritoque (32°49′ S, 71°31′ W), Quintay (33°10′ S, 71°41′ W), Topocalma (34°06′ S, 71°58′ W), and Pichilemu (34°26′ S, 72°02′ W).

For sampling reference populations of the parental species *B. macraei* and *B. linearis*, areas were selected where only one of these species was found and where there was no morphological evidence of hybridization. Specifically, *B. macraei* was sampled at the coast near Huentelauquén (31°36′ S, 71° 33′ W), Papudo (32°29′ S, 71°25′ W), and Navidad (33°56′ S, 71°50′ W), while *B. linearis* was sampled inland near Cavilolén (31°46′ S, 71°19′ W), Río Petorca (33°22′ S, 71°07′ W), Quinicabén (34°01′ S, 71°43′ W), and Catapilco (32°34′ S, 71°15′ W). For each reference population, 30 plants were sampled as described above for the transects.

During the second field trip in September 2022, a subset of the plants already labeled and sampled in the previous field trip in Pichidangui and Quintay (hybridization zones), Papudo and Navidad (*B. macraei* reference populations), and Cavilolén and Quinicabén (*B. linearis* reference populations) were resampled to identify the effects of the season on the plant metabolomes.

### Establishment of a greenhouse population

In March 2019, seeds were collected from plant individuals of hybrid swarm populations in two different locations. Specifically, seeds were obtained from plants morphologically classified as *B. × intermedia* (one individual from Pichidangui), *B. macraei* (six individuals from Pichidangui), and *B. linearis* (two individuals from Catapilco). Collected seeds were sown and grown in the greenhouse of the Jena Botanical Garden. Leaves of 14 plants (1 year old) from each species of the different lines were collected, flash‐frozen in liquid nitrogen, and stored at −80°C for subsequent analyses of volatile and non‐volatile leaf metabolites. In contrast to the field sampling in Chile, where the leaf material was dried with silica gel, resulting in a loss of volatile compounds, leaves harvested in the greenhouse were freshly frozen and powdered under liquid nitrogen. This procedure allowed not only the extraction of metabolites with methanol for untargeted metabolomics (LC‐qTOF‐MS), but also the extraction of volatile compounds with hexane for gas chromatography–mass spectrometry (GC‐MS). Note that the progeny of the *B. × intermedia* plant represents either an F2 generation if the maternal plant is assumed to be a primary (F1) hybrid or an even later generation if the maternal plant belongs to a later generation (F > 2 or greater). Progeny of plants identified as *B. linearis* and *B. macraei* may have originated from either intra‐ or interspecific crosses.

### DNA extraction and genotyping by sequencing (GBS)

Leaf tissue samples were sent to LGC Genomics GmbH in Berlin for DNA extraction and sequencing in dried plate format. All DNA extractions were eluted in Tris buffer containing EDTA (10 mM Tris, 0.1 mM EDTA). DNA was isolated using the sbeadexTM mini plant kit according to the LGC Plant Kit 2017 protocol. Lysis buffer was additionally mixed with 1% thioglycerol and RNase. Genotyping by sequencing (GBS) was used to generate genome‐wide polymorphism data (Elshire et al., 2011). The library for GBS was prepared using the enzyme combination *Pst*I‐*Ape*KI, and pooling was performed for 2 × 150 bp sequences on the Illumina NextSeq 500/550 v2. The LGC library preparation protocol 2019 was used for library preparation. Sequencing was targeted at an average of 1.0 million reads per sample. The average insert size range was approximately 190 bp. A total of 384 million raw reads were obtained for the entire dataset. Pre‐processing was performed in several steps. (1) All library groups were demultiplexed using the Illumina bcl2fastq 2.20 software. (2) One or two mismatches or Ns were allowed in the barcode read if the barcode distances between all libraries on the lane allowed it. Also, no mismatches or Ns were allowed in the inline barcodes, but Ns were allowed in the restriction site. (3) Sequencing adapter residues were removed from all reads. (4) Reads with a final length < 20 bases were discarded. (5) Restriction enzyme site filtering at 5' of read ends. (6) Quality trimming of Illumina restriction enzyme reads. (7) Removal of reads containing Ns. (8) Trimming of reads at the 3′ end to obtain an average Phred quality score of at least 20 over a window of 10 bases. (9) Reads with a final length < 20 bases were discarded. After these steps, the combined reads were clustered using CD‐HIT‐EST, allowing up to 5% difference (Huang et al., 2010). Clusters were filtered to exclude singletons and clusters formed from <20 reads. Alignment of the subsampled quality trimmed reads to the reference sequence was performed using BWA‐MEM v. 0.7.12 software. In the absence of a complete *Baccharis* genome, the “artificia” reference sequence generated and described in Schneider and Hellwig (2024) was used as a reference. Variant detection and genotyping of samples were performed using Freebayes v1.2.0 software (Garrison & Marth, 2012). The following specific parameters were used: min base quality = 10, min supporting allele qsum = 10, read mismatch limit = 3, min coverage = 5, no indels, min alternative count = 4, exclude unobserved genotypes, ploidy = 2, no mnps, no complexes, mismatch base quality threshold = 10. Variants were filtered using a GBS‐specific rule set: (1) The number of reads for a locus must exceed 8 reads. (2) Genotypes must be observed in at least 64 of the samples. (3) The minimum allele frequency across all samples must exceed 5%. The data were stored in variant call format v. 4.2 (vcf, Danecek et al., 2011). GBS results are summarized in Figure S1. After clustering of genetic sequences, the number of clustered loci was 63 348 with a mapping rate of 74.3%. A total of 30 773 polymorphic loci were counted, with a total number of 120 064 SNPs across all samples. Filtering of the variants resulted in 4463 SNPs, which were used for further population genetic analysis.

### Untargeted metabolomics by UHPLC–ESI–HRMS

For analysis of the plant material collected in Chile, approximately 0.3 g of dried leaves were weighed and extracted using 4 mL methanol. Material was ground in the methanolic solution by adding three metal beads (3.5 mm) during 15 min using a paint‐shaker (Skandex SO‐10 M, Fluid Management Europe, The Netherlands). Samples were centrifuged at room temperature for 15 min at 15,000 ***g***, diluted 1:200, and analyzed in randomized order using ultra‐high‐performance liquid chromatography–electrospray ionization–high‐resolution mass spectrometry (UHPLC–ESI–HRMS), inserting quality controls every 20 samples. UHPLC–ESI–HRMS was performed with a Dionex Ultimate 3000 series UHPLC (Thermo Scientific) and a Bruker timsToF mass spectrometer (Bruker Daltonics, Bremen, Germany). UHPLC was used by applying a reversed‐phase Zorbax Eclipse XDB‐C18 column (100 mm × 2.1 mm, 1.8 μm, Agilent Technologies, Waldbronn, Germany) with a solvent system of 0.1% formic acid (A) and acetonitrile (B) at a flow rate of 0.3 mL/min. The elution profile was the following: 0–0.5 min, 30% B; 0.5–11.0 min, 30%–90% B; 11.0–11.1 min, 90%–100% B; 11.1–12.0 min, 100% B; and 12.1–15.0 min, 30% B. Electrospray ionization (ESI) in negative/positive ionization mode was used for the coupling of LC to MS. The mass spectrometer parameters were set as follows: capillary voltage 4.5 KV/3.5 KV, end plate offset of 500 V, nebulizer pressure 2.8 bar, nitrogen at 280°C at a flow rate of 8 L/min as drying gas. Acquisition was achieved at 12 Hz with a mass range from *m*/*z* 50 to 1500, with data‐dependent MS/MS and an active exclusion window of 0.1 min, a reconsideration threshold of 1.8‐fold change, and an exclusion after five spectra. Fragmentation was triggered on an absolute threshold of 50 counts and acquired on the two most intense peaks with MS/MS spectra acquisition of 12 Hz. Collision energy was alternated between 20 and 50 V. At the beginning of each chromatographic analysis, 10 μL of a sodium formate‐isopropanol solution (10 mM solution of NaOH in 50/50 (*v*/*v*%) isopropanol water containing 0.2% formic acid) was injected into the dead volume of the sample injection for re‐calibration of the mass spectrometer using the expected cluster ion *m*/*z* values.

For the analysis of the greenhouse plants, approximately 40 mg of frozen and ground plant tissue was extracted with 800 μL methanol and incubated for 30 min at room temperature under continuous shaking. Supernatant was recovered, and samples were analyzed using UHPLC–ESI–HRMS as described above.

All data are given in supplemental datasets SI [Link], [Link], [Link], [Link].

### Hexane extraction and GC‐MS/GC‐FID analysis

Frozen leaf samples from the greenhouse plants were ground, and approximately 40 mg of plant material was extracted for 2 h at room temperature under continuous shaking using 400 μL hexane containing 10 ng/μL nonyl acetate as an internal standard. Note: Nonyl acetate could not be detected in extracts produced with pure hexane, which shows that the *Baccharis* species analyzed are not capable of producing nonyl acetate naturally. Samples were centrifuged for 5 min at 4000 ***g***, and supernatants were qualitatively analyzed with gas chromatography–mass spectrometry (GC‐MS) and quantitatively analyzed with gas chromatography–flame ionization detection (GC‐FID). Specifically, samples were analyzed using a 6890 Series gas chromatograph (Agilent Technologies, Santa Clara, CA, USA) coupled to an Agilent 5973 quadrupole mass selective detector (interface temp, 270°C; quadrupole temp, 150°C; source temp, 230°C; electron energy, 70 eV) or a flame ionization detector (FID) operated at 300°C, respectively. For the separation of mono‐ and sesquiterpenes using a ZB5 column (Phenomenex, Aschaffenburg, Germany, 30 m × 0.25 mm × 0.25 μm) and He (MS) or H_2_ (FID) as carrier gas, 1 μL of sample was injected without split at an initial oven temperature of 45°C, the temperature was held for 2 min, and then increased to 300°C with a gradient of 6°C min^−1^, and then further increased to 320°C with a gradient of 60°C min^−1^ and a hold of 2 min. For diterpene analysis, the initial oven temperature was 100°C, the temperature was held for 2 min and then increased to 310°C with a gradient of 7°C min^−1^, and further increased to 320°C with a gradient of 60°C min^−1^ and a hold of 2 min. GC‐FID quantification data and peak identification using GC‐MS were manually combined, and quantification was corrected to the internal standard intensity and sample weight. Compounds were identified by comparing their retention times and mass spectra to those of authentic standards or to reference spectra in the Wiley and National Institute of Standards and Technology (NIST) Libraries.

### Genetic data analysis

To reveal the genetic structure in the dataset, we used principal component analysis (PCA) and sparse non‐negative matrix factorization (sNMF). For the PCA, the .vcf file was converted into a .geno file and an .indv file using pdg‐spider, version 2.1.1.5v (Lischer & Excoffier, 2011). PCA and plotting were performed in R, version 4.3.1, using the package LEA, v. 3.14.0 for PCA (Francois, 2015) and the package ggplot2, v. 3.4.4 (Wickham, 2016) for plotting. To further explore the genetic substructure of the dataset (246 individuals, 4463 SNPs), samples were clustered using a non‐spatial, non‐Bayesian approach provided by the LEA package v. 3.14.0, in R v. 4.3.1 (Francois, 2015). sNMF was favored against other Bayesian methods such as the “structure” or “TESS 2.3” algorithms (Frichot et al., 2014), because of its robustness to deviations from population genetic models and its superior speed. Clustering was performed with different numbers of clusters (1–20), a regularization parameter α = 5, a tolerance parameter ε = 10^−4^, 5% of masked genotypes when calculating the cross‐entropy criterion, and 100 replicates per cluster. Model fitting and estimation of the number of clusters are based on the cross‐entropy value (Eastment & Krzanowski, 1982; Frichot et al., 2014; Wold, 1978). CLUMPAK was used to summarize the different runs (Kopelman et al., 2015). The best run of the selected clusters was visualized using the packages pophelper v. 2.3.1 (Francis, 2017), dplyr v. 1.1.3 (Vaughan et al., 2023), and ggplot2 v. 3.4.4 (Wickham, 2016).

### LC‐MS and GC‐MS/FID data processing and analysis

LC‐MS peak detection was carried out using Metaboscape software (Bruker Daltonik, Bremen, Germany) with the T‐Rex 3D algorithm for qTOF data. For peak detection, an intensity threshold of 1000 with a minimum of 10 spectra and a time window from 0.4 to 11 min was used. Adducts of [M + H]^+^, [M + Na]^+^, [M + K]^+^, and [2 M + H]^+^ (for positive mode) or [M‐H]^–^, [M + Cl]^–^, and [M + COOH]^–^ (for negative mode) were grouped as a single bucket if they had an EIC correlation of 0.8. The signal intensity of a bucket is equal to the intensity of the feature with the highest intensity among those assigned to the same bucket. Because in‐source fragmentation, which is often seen especially in positive ionization mode, was not considered in the bucketing process, features with the same retention time may represent different in‐source fragments from the same compound. Results were exported to GNPS for Feature‐Based Molecular Networking (FBMN) workflow analysis (Nothias et al., 2020). The data were filtered by removing all MS/MS fragment ions within +/− 17 Da of the precursor *m/z*. MS/MS spectra were window‐filtered by choosing only the top six fragment ions in the +/− 50 Da window throughout the spectrum. The precursor ion mass tolerance was set to 0.02 Da and the MS/MS fragment ion tolerance to 0.02 Da. A molecular network was then created where edges were filtered to have a cosine score above 0.7 and more than six matched peaks. Further, edges between two nodes were kept in the network if and only if each of the nodes appeared in each other's respective top 10 most similar nodes. Finally, the maximum size of a molecular family was set to 100, and the lowest scoring edges were removed from molecular families until the molecular family size was below this threshold. The spectra in the network were then searched against GNPS spectral libraries (Horai et al., 2010; Wang et al., 2016). The library spectra were filtered in the same manner as the input data. All matches kept between network spectra and library spectra were required to have a score above 0.7 and at least six matched peaks. The DEREPLICATOR was used to annotate MS/MS spectra (Mohimani et al., 2018). The molecular networks were visualized using Cytoscape software (Shannon et al., 2003). Mass spectral data were annotated with chemical superclass, class, and subclass according to SIRIUS (Djoumbou Feunang et al., 2016; Dührkop et al., 2019, 2021), using default parameters. By combining SIRIUS annotation and the GNPS FBMN, groups of features with similar chemical class annotation as well as differences between the two different species metabolic profiles were identified. PCA was performed in R using R base function prcomp and ggplot2 v. 3.4.4 (Wickham, 2016). Statistical analysis (ANOVA and Tukey's test) was performed using the R package multicomp v. 1.4–28 (Hothorn et al., 2008). For metabolome analysis, PCA was always performed with data from the positive ionization mode. To determine the 20 LC‐MS features that contribute the most to species separation (see Table 1), we examined their contribution to the first principal component (PC1). Specifically, we extracted the contribution of each variable to PC1 using the get_pca_var() function and ordered them from most to least important. Metabolic diversity was calculated as (a) the number of metabolites within a plant individual (richness of specialized metabolites) and (b) the abundance‐weighted diversity of metabolites expressed as the Shannon–Weaver index based on plant individual‐level metabolite intensities (Hill, 1973) using the R package vegan.

### Isolation and NMR analysis of diterpenoids

Bacchasmacranone (Gambaro et al., 1986), hautriwaic acid (Hsü et al., 1971; Huang et al., 2010), 1‐deoxybacrispine (Ceñal et al., 1997), 2β‐hydroxy‐hautriwaic lactone (Maldonado et al., 1996), along with two new neo‐clerodane diterpenes, 8‐*epi*‐bacchasmacranone and 18‐dihydro‐conyscabrafuran, were isolated from *B. macraei* leaf material and identified by high‐resolution ESI mass spectrometry and NMR analysis as described in detail in supplemental dataset SI5. Hardwickiic acid was purchased from Wuhan ChemNorm Biotech (Wuhan, China).

## Author Contributions

OZD, FS, FHH, and TGK performed the field sampling. OZD, MR, and YN performed LC‐MS analysis. OZD performed the GC‐MS analyses, analyzed the data, and drafted the manuscript. FS provided the genetic characterization of the plant samples. YN isolated diterpenoids and performed NMR analysis. TGK, FHH, and JG designed the study, coordinated and supervised the project, and edited the manuscript. All authors read and approved the manuscript.

## Conflict of Interest Statement

The authors declare that they have no competing interests.

## Supporting information


**Dataset S1.** Metadata for *Baccharis* samples collected in March 2022.

**Dataset S2.** Relative quantification (peak area) of LC‐MS‐MS data from *Baccharis* samples collected in March 2022.

**Dataset S3.** Metadata for *Baccharis* samples collected in September 2022.

**Dataset S4.** Relative quantification (peak area) of LC‐MS‐MS data from *Baccharis* samples collected in September 2022.

**Dataset S5.** Isolation and compound characterization of diterpenoids.

**Figure S1.** GBS results. Group: all samples, reads were mapped against the reference created in (Schneider & Hellwig, 2024).
**Figure S2.** Principal component analysis based on the profiles of semi‐polar metabolites, obtained by LC‐MS measurements, for the samples morphologically characterized as *B. macraei*, *B. linearis*, and *B. × intermedia*.
**Figure S3.** Genetic cluster assignments (A, *K* = 3; B, *K* = 6) of the investigated *Baccharis* taxa using LEA v. 2.6.0 shown as barplots.
**Figure S4.** Examples of molecular networks containing features abundant in either (A) *B. macraei* and *B. × intermedia* or (B) *B. linearis* and *B. × intermedia*. Each node corresponds to a metabolic feature, edges present when the cosine similarity between spectra is >0.7.
**Figure S5.** Molecular network containing compounds annotated as flavonoids. Each node corresponds to a metabolic feature, edges present when the cosine similarity between spectra is >0.7. Yellow, *B. linearis*; green, *B. × intermedia*; blue, *B. macraei*.
**Figure S6.** Molecular network containing compounds annotated as diterpenoids. Each node corresponds to a metabolic feature, edges present when the cosine similarity between spectra is >0.7. Yellow, *B. linearis*; green, *B. × intermedia*; blue, *B. macraei*. Black circles indicate a high abundance.
**Figure S7.** Molecular network containing compounds annotated as triterpenoids. Each node corresponds to a metabolic feature, edges present when the cosine similarity between spectra is >0.7. Yellow, *B. linearis*; green, *B. × intermedia*; blue, *B. macraei*. Black circles indicate a high abundance.
**Figure S8.** Pie‐charts summarizing the chemical superclass annotation of (A) metabolic features classified as primary metabolites and (B) metabolic features classified as secondary metabolites. The numbers represent the number of features in each chemical class.
**Figure S9.** Principal component analysis based on the profiles of the metabolic features annotated as primary metabolites, colored according the location (ordered from North to South).
**Figure S10.** Relative abundance of (A) hardwickiic acid, (B) hautriwaic acid, (C) 2β‐hydroxy hautriwaic lactone, (D) bacchasmacranone, (E) 17‐*epi*‐bacchasmacranone, (F) 18‐dihydro‐conyscabrafuran, and (G) 1‐deoxybacrispine in *B. linearis*, *B. macraei, and B. × intermedia*.
**Figure S11.** Principal component analysis based on the metabolomes obtained by LC‐MS measurements of methanolic leaf extracts, colored according to (A) the sex of the plant, and (B) the season of sample collection.

## Data Availability

SNP data, including metadata, are available at https://doi.org/10.17617/3.MWU9ML. LC‐MS data were stored as .mzML files in the EDMOND data archive of the Max Planck Society and are available at https://doi.org/10.17617/3.BKO7BY.
