# Supplementary material for: Consequences of interspecific plant hybridization on metabolic diversity in naturally occurring hybrid swarms
Source: Plant J. 2025 Aug 26;123(4):e70444. doi: 10.1111/tpj.70444 (PMC12380477; doi:10.1111/tpj.70444)
Supplement: Supplementary file 5 — Dataset S5. Isolation and compound characterization of diterpenoids. [file TPJ-123-0-s005.pptx]

## Slide 1
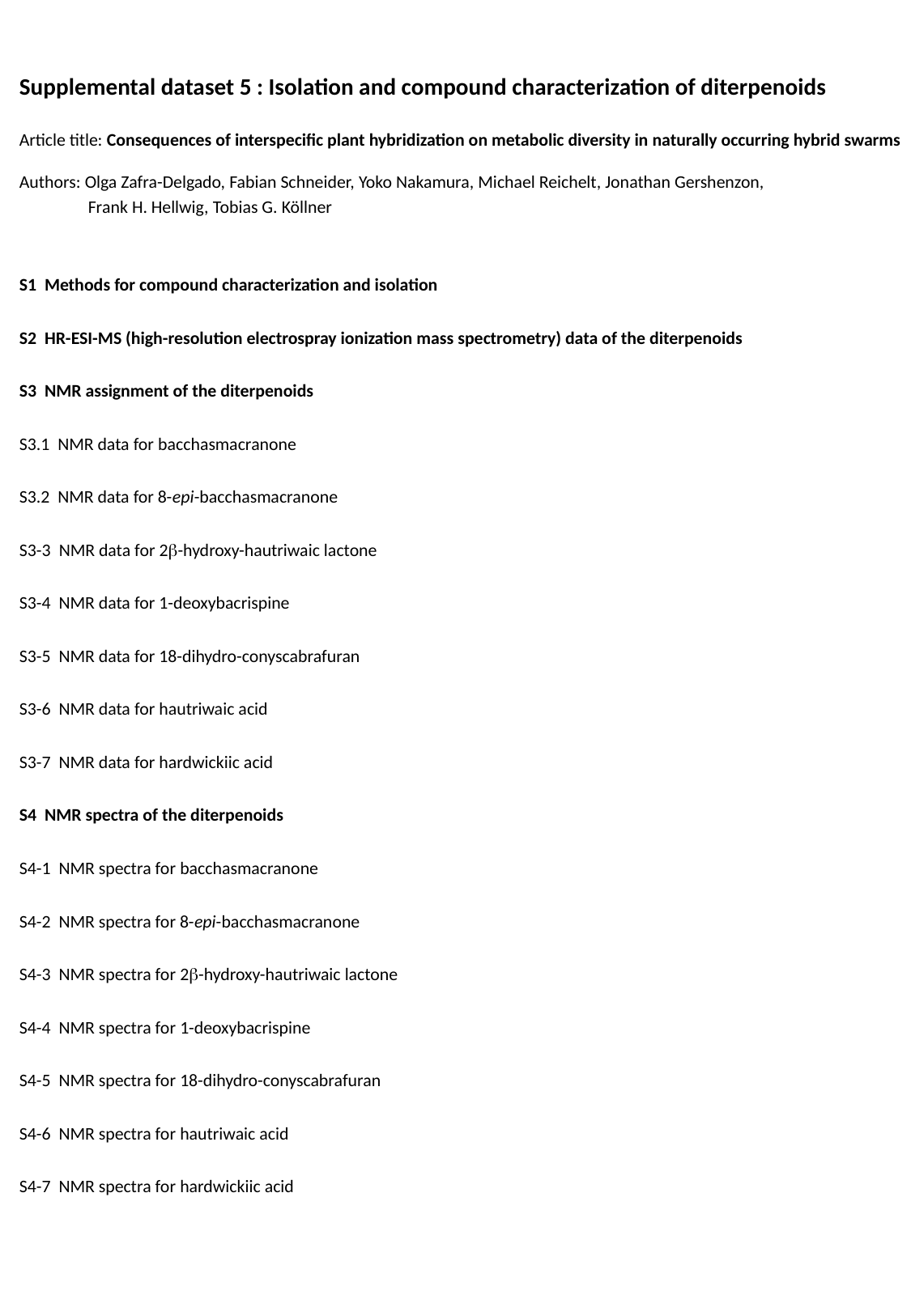

Supplemental dataset 5 : Isolation and compound characterization of diterpenoids
Article title: Consequences of interspecific plant hybridization on metabolic diversity in naturally occurring hybrid swarms
Authors: Olga Zafra-Delgado, Fabian Schneider, Yoko Nakamura, Michael Reichelt, Jonathan Gershenzon,
 Frank H. Hellwig, Tobias G. Köllner
S1 Methods for compound characterization and isolation
S2 HR-ESI-MS (high-resolution electrospray ionization mass spectrometry) data of the diterpenoids
S3 NMR assignment of the diterpenoids
S3.1 NMR data for bacchasmacranone
S3.2 NMR data for 8-epi-bacchasmacranone
S3-3 NMR data for 2b-hydroxy-hautriwaic lactone
S3-4 NMR data for 1-deoxybacrispine
S3-5 NMR data for 18-dihydro-conyscabrafuran
S3-6 NMR data for hautriwaic acid
S3-7 NMR data for hardwickiic acid
S4 NMR spectra of the diterpenoids
S4-1 NMR spectra for bacchasmacranone
S4-2 NMR spectra for 8-epi-bacchasmacranone
S4-3 NMR spectra for 2b-hydroxy-hautriwaic lactone
S4-4 NMR spectra for 1-deoxybacrispine
S4-5 NMR spectra for 18-dihydro-conyscabrafuran
S4-6 NMR spectra for hautriwaic acid
S4-7 NMR spectra for hardwickiic acid

## Slide 2
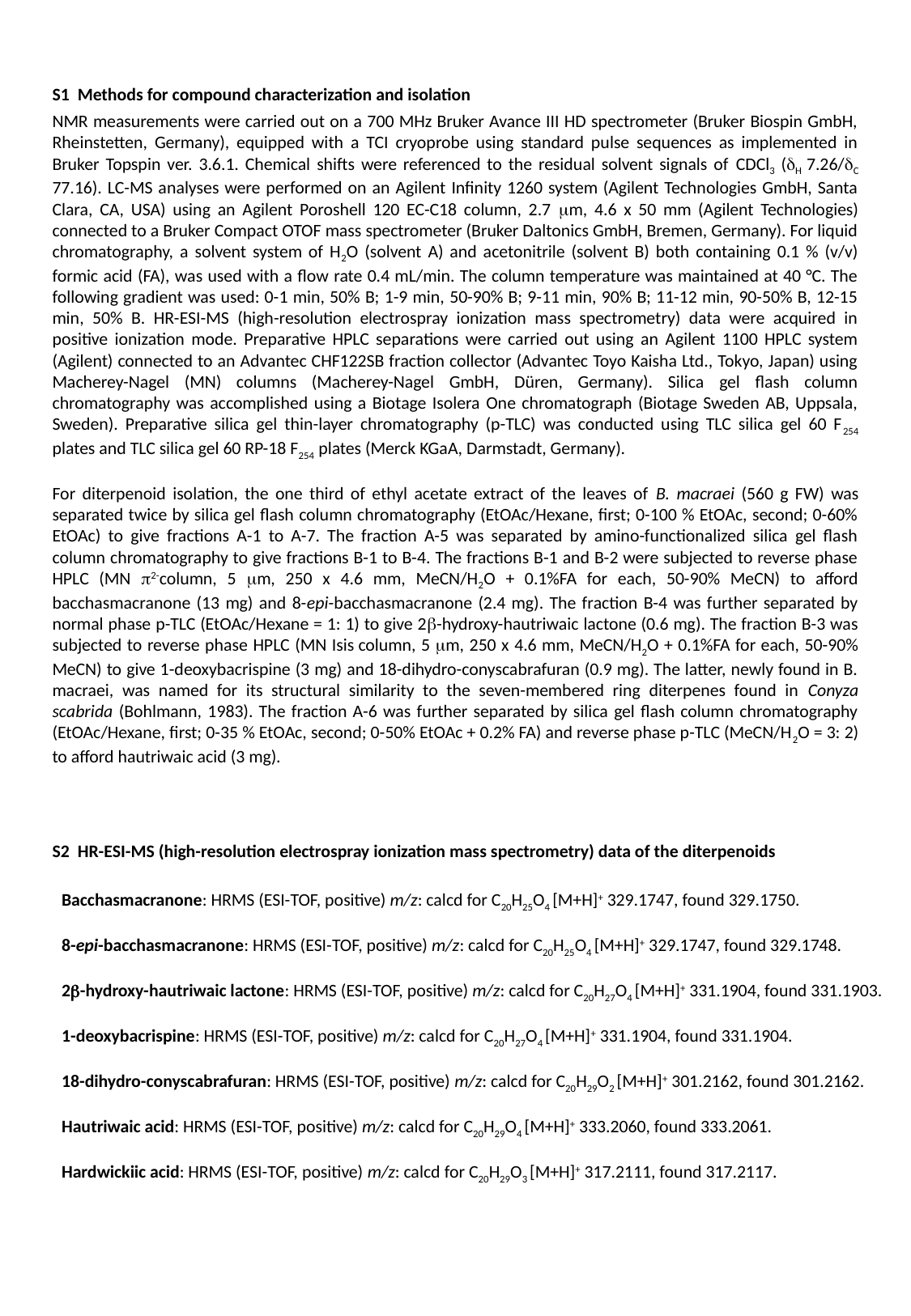

S1 Methods for compound characterization and isolation
NMR measurements were carried out on a 700 MHz Bruker Avance III HD spectrometer (Bruker Biospin GmbH, Rheinstetten, Germany), equipped with a TCI cryoprobe using standard pulse sequences as implemented in Bruker Topspin ver. 3.6.1. Chemical shifts were referenced to the residual solvent signals of CDCl3 (dH 7.26/dC 77.16). LC-MS analyses were performed on an Agilent Infinity 1260 system (Agilent Technologies GmbH, Santa Clara, CA, USA) using an Agilent Poroshell 120 EC-C18 column, 2.7 mm, 4.6 x 50 mm (Agilent Technologies) connected to a Bruker Compact OTOF mass spectrometer (Bruker Daltonics GmbH, Bremen, Germany). For liquid chromatography, a solvent system of H2O (solvent A) and acetonitrile (solvent B) both containing 0.1 % (v/v) formic acid (FA), was used with a flow rate 0.4 mL/min. The column temperature was maintained at 40 °C. The following gradient was used: 0-1 min, 50% B; 1-9 min, 50-90% B; 9-11 min, 90% B; 11-12 min, 90-50% B, 12-15 min, 50% B. HR-ESI-MS (high-resolution electrospray ionization mass spectrometry) data were acquired in positive ionization mode. Preparative HPLC separations were carried out using an Agilent 1100 HPLC system (Agilent) connected to an Advantec CHF122SB fraction collector (Advantec Toyo Kaisha Ltd., Tokyo, Japan) using Macherey-Nagel (MN) columns (Macherey-Nagel GmbH, Düren, Germany). Silica gel flash column chromatography was accomplished using a Biotage Isolera One chromatograph (Biotage Sweden AB, Uppsala, Sweden). Preparative silica gel thin-layer chromatography (p-TLC) was conducted using TLC silica gel 60 F254 plates and TLC silica gel 60 RP-18 F254 plates (Merck KGaA, Darmstadt, Germany).
For diterpenoid isolation, the one third of ethyl acetate extract of the leaves of B. macraei (560 g FW) was separated twice by silica gel flash column chromatography (EtOAc/Hexane, first; 0-100 % EtOAc, second; 0-60% EtOAc) to give fractions A-1 to A-7. The fraction A-5 was separated by amino-functionalized silica gel flash column chromatography to give fractions B-1 to B-4. The fractions B-1 and B-2 were subjected to reverse phase HPLC (MN p2-column, 5 mm, 250 x 4.6 mm, MeCN/H2O + 0.1%FA for each, 50-90% MeCN) to afford bacchasmacranone (13 mg) and 8-epi-bacchasmacranone (2.4 mg). The fraction B-4 was further separated by normal phase p-TLC (EtOAc/Hexane = 1: 1) to give 2b-hydroxy-hautriwaic lactone (0.6 mg). The fraction B-3 was subjected to reverse phase HPLC (MN Isis column, 5 mm, 250 x 4.6 mm, MeCN/H2O + 0.1%FA for each, 50-90% MeCN) to give 1-deoxybacrispine (3 mg) and 18-dihydro-conyscabrafuran (0.9 mg). The latter, newly found in B. macraei, was named for its structural similarity to the seven-membered ring diterpenes found in Conyza scabrida (Bohlmann, 1983). The fraction A-6 was further separated by silica gel flash column chromatography (EtOAc/Hexane, first; 0-35 % EtOAc, second; 0-50% EtOAc + 0.2% FA) and reverse phase p-TLC (MeCN/H2O = 3: 2) to afford hautriwaic acid (3 mg).
S2 HR-ESI-MS (high-resolution electrospray ionization mass spectrometry) data of the diterpenoids
Bacchasmacranone: HRMS (ESI-TOF, positive) m/z: calcd for C20H25O4 [M+H]+ 329.1747, found 329.1750.
8-epi-bacchasmacranone: HRMS (ESI-TOF, positive) m/z: calcd for C20H25O4 [M+H]+ 329.1747, found 329.1748.
2b-hydroxy-hautriwaic lactone: HRMS (ESI-TOF, positive) m/z: calcd for C20H27O4 [M+H]+ 331.1904, found 331.1903.
1-deoxybacrispine: HRMS (ESI-TOF, positive) m/z: calcd for C20H27O4 [M+H]+ 331.1904, found 331.1904.
18-dihydro-conyscabrafuran: HRMS (ESI-TOF, positive) m/z: calcd for C20H29O2 [M+H]+ 301.2162, found 301.2162.
Hautriwaic acid: HRMS (ESI-TOF, positive) m/z: calcd for C20H29O4 [M+H]+ 333.2060, found 333.2061.
Hardwickiic acid: HRMS (ESI-TOF, positive) m/z: calcd for C20H29O3 [M+H]+ 317.2111, found 317.2117.

## Slide 3
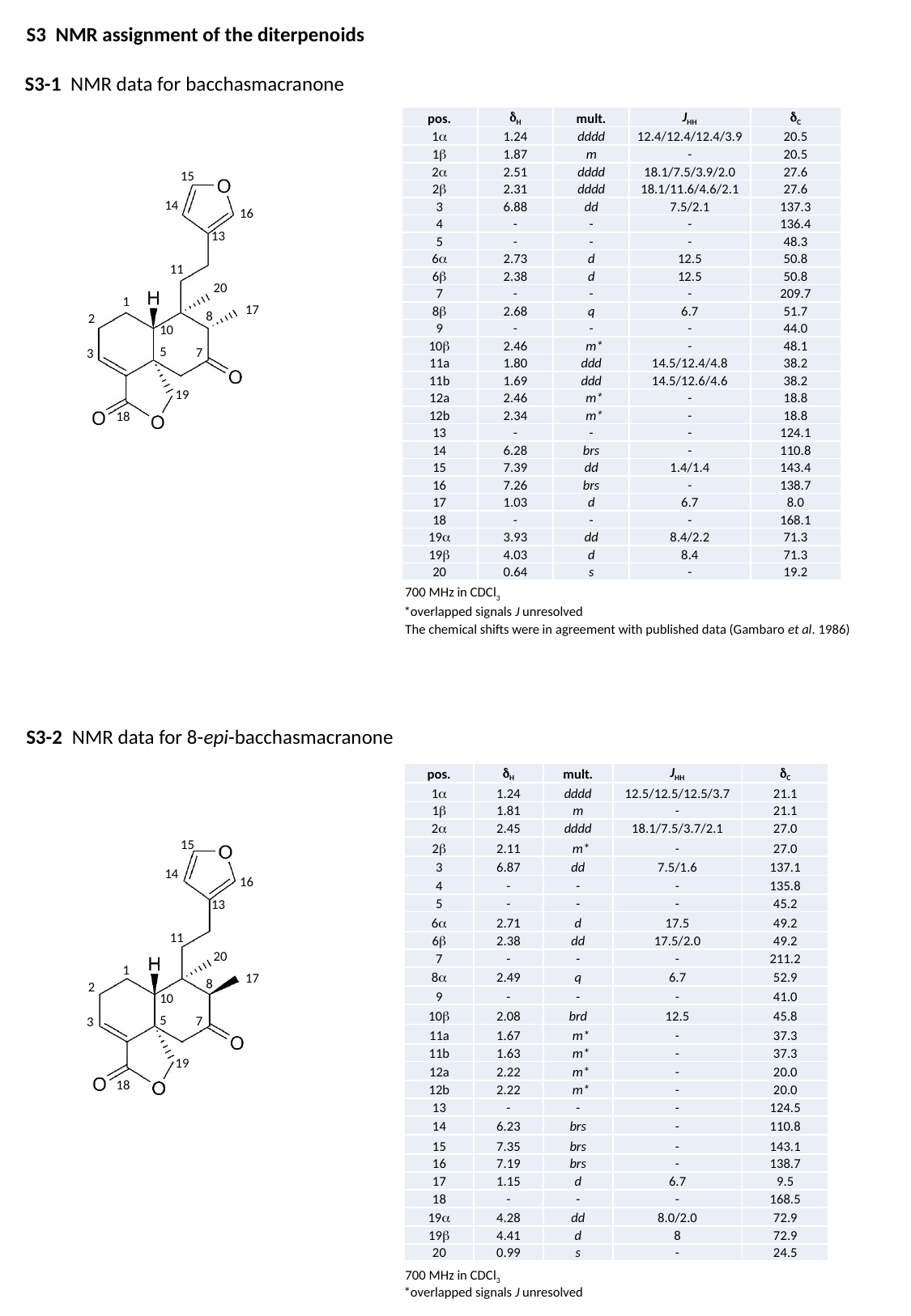

S3 NMR assignment of the diterpenoids
S3-1 NMR data for bacchasmacranone
| pos. | dH | mult. | JHH | dC |
| --- | --- | --- | --- | --- |
| 1a | 1.24 | dddd | 12.4/12.4/12.4/3.9 | 20.5 |
| 1b | 1.87 | m | - | 20.5 |
| 2a | 2.51 | dddd | 18.1/7.5/3.9/2.0 | 27.6 |
| 2b | 2.31 | dddd | 18.1/11.6/4.6/2.1 | 27.6 |
| 3 | 6.88 | dd | 7.5/2.1 | 137.3 |
| 4 | - | - | - | 136.4 |
| 5 | - | - | - | 48.3 |
| 6a | 2.73 | d | 12.5 | 50.8 |
| 6b | 2.38 | d | 12.5 | 50.8 |
| 7 | - | - | - | 209.7 |
| 8b | 2.68 | q | 6.7 | 51.7 |
| 9 | - | - | - | 44.0 |
| 10b | 2.46 | m\* | - | 48.1 |
| 11a | 1.80 | ddd | 14.5/12.4/4.8 | 38.2 |
| 11b | 1.69 | ddd | 14.5/12.6/4.6 | 38.2 |
| 12a | 2.46 | m\* | - | 18.8 |
| 12b | 2.34 | m\* | - | 18.8 |
| 13 | - | - | - | 124.1 |
| 14 | 6.28 | brs | - | 110.8 |
| 15 | 7.39 | dd | 1.4/1.4 | 143.4 |
| 16 | 7.26 | brs | - | 138.7 |
| 17 | 1.03 | d | 6.7 | 8.0 |
| 18 | - | - | - | 168.1 |
| 19a | 3.93 | dd | 8.4/2.2 | 71.3 |
| 19b | 4.03 | d | 8.4 | 71.3 |
| 20 | 0.64 | s | - | 19.2 |
15
14
16
13
11
20
1
17
8
2
10
5
7
3
19
18
700 MHz in CDCl3
*overlapped signals J unresolved
The chemical shifts were in agreement with published data (Gambaro et al. 1986)
S3-2 NMR data for 8-epi-bacchasmacranone
| pos. | dH | mult. | JHH | dC |
| --- | --- | --- | --- | --- |
| 1a | 1.24 | dddd | 12.5/12.5/12.5/3.7 | 21.1 |
| 1b | 1.81 | m | - | 21.1 |
| 2a | 2.45 | dddd | 18.1/7.5/3.7/2.1 | 27.0 |
| 2b | 2.11 | m\* | - | 27.0 |
| 3 | 6.87 | dd | 7.5/1.6 | 137.1 |
| 4 | - | - | - | 135.8 |
| 5 | - | - | - | 45.2 |
| 6a | 2.71 | d | 17.5 | 49.2 |
| 6b | 2.38 | dd | 17.5/2.0 | 49.2 |
| 7 | - | - | - | 211.2 |
| 8a | 2.49 | q | 6.7 | 52.9 |
| 9 | - | - | - | 41.0 |
| 10b | 2.08 | brd | 12.5 | 45.8 |
| 11a | 1.67 | m\* | - | 37.3 |
| 11b | 1.63 | m\* | - | 37.3 |
| 12a | 2.22 | m\* | - | 20.0 |
| 12b | 2.22 | m\* | - | 20.0 |
| 13 | - | - | - | 124.5 |
| 14 | 6.23 | brs | - | 110.8 |
| 15 | 7.35 | brs | - | 143.1 |
| 16 | 7.19 | brs | - | 138.7 |
| 17 | 1.15 | d | 6.7 | 9.5 |
| 18 | - | - | - | 168.5 |
| 19a | 4.28 | dd | 8.0/2.0 | 72.9 |
| 19b | 4.41 | d | 8 | 72.9 |
| 20 | 0.99 | s | - | 24.5 |
15
14
16
13
11
20
1
17
8
2
10
5
7
3
19
18
700 MHz in CDCl3
*overlapped signals J unresolved

## Slide 4
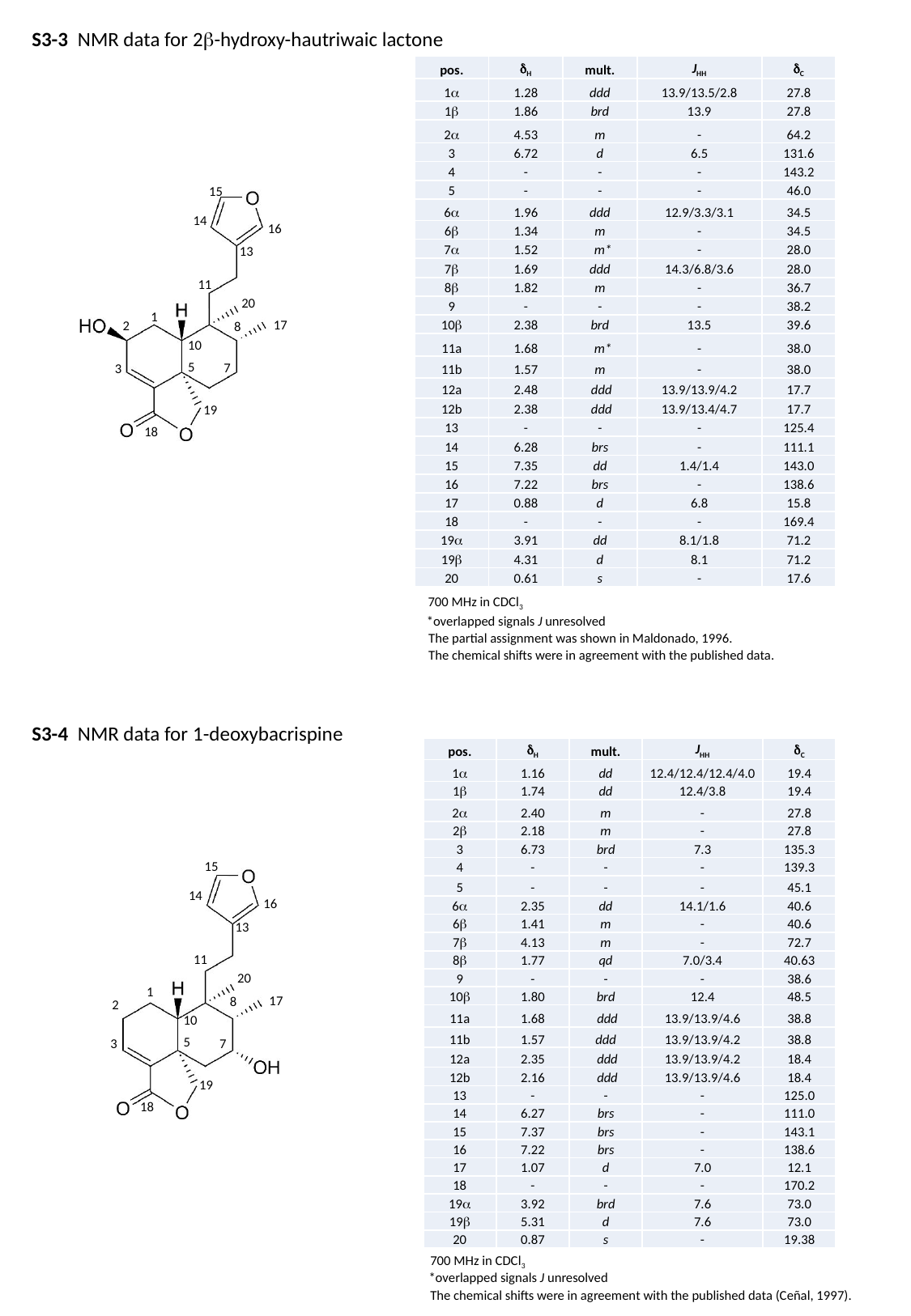

S3-3 NMR data for 2b-hydroxy-hautriwaic lactone
| pos. | dH | mult. | JHH | dC |
| --- | --- | --- | --- | --- |
| 1a | 1.28 | ddd | 13.9/13.5/2.8 | 27.8 |
| 1b | 1.86 | brd | 13.9 | 27.8 |
| 2a | 4.53 | m | - | 64.2 |
| 3 | 6.72 | d | 6.5 | 131.6 |
| 4 | - | - | - | 143.2 |
| 5 | - | - | - | 46.0 |
| 6a | 1.96 | ddd | 12.9/3.3/3.1 | 34.5 |
| 6b | 1.34 | m | - | 34.5 |
| 7a | 1.52 | m\* | - | 28.0 |
| 7b | 1.69 | ddd | 14.3/6.8/3.6 | 28.0 |
| 8b | 1.82 | m | - | 36.7 |
| 9 | - | - | - | 38.2 |
| 10b | 2.38 | brd | 13.5 | 39.6 |
| 11a | 1.68 | m\* | - | 38.0 |
| 11b | 1.57 | m | - | 38.0 |
| 12a | 2.48 | ddd | 13.9/13.9/4.2 | 17.7 |
| 12b | 2.38 | ddd | 13.9/13.4/4.7 | 17.7 |
| 13 | - | - | - | 125.4 |
| 14 | 6.28 | brs | - | 111.1 |
| 15 | 7.35 | dd | 1.4/1.4 | 143.0 |
| 16 | 7.22 | brs | - | 138.6 |
| 17 | 0.88 | d | 6.8 | 15.8 |
| 18 | - | - | - | 169.4 |
| 19a | 3.91 | dd | 8.1/1.8 | 71.2 |
| 19b | 4.31 | d | 8.1 | 71.2 |
| 20 | 0.61 | s | - | 17.6 |
15
14
16
13
11
20
1
17
2
8
10
5
7
3
19
18
700 MHz in CDCl3
*overlapped signals J unresolved
The partial assignment was shown in Maldonado, 1996.
The chemical shifts were in agreement with the published data.
S3-4 NMR data for 1-deoxybacrispine
| pos. | dH | mult. | JHH | dC |
| --- | --- | --- | --- | --- |
| 1a | 1.16 | dd | 12.4/12.4/12.4/4.0 | 19.4 |
| 1b | 1.74 | dd | 12.4/3.8 | 19.4 |
| 2a | 2.40 | m | - | 27.8 |
| 2b | 2.18 | m | - | 27.8 |
| 3 | 6.73 | brd | 7.3 | 135.3 |
| 4 | - | - | - | 139.3 |
| 5 | - | - | - | 45.1 |
| 6a | 2.35 | dd | 14.1/1.6 | 40.6 |
| 6b | 1.41 | m | - | 40.6 |
| 7b | 4.13 | m | - | 72.7 |
| 8b | 1.77 | qd | 7.0/3.4 | 40.63 |
| 9 | - | - | - | 38.6 |
| 10b | 1.80 | brd | 12.4 | 48.5 |
| 11a | 1.68 | ddd | 13.9/13.9/4.6 | 38.8 |
| 11b | 1.57 | ddd | 13.9/13.9/4.2 | 38.8 |
| 12a | 2.35 | ddd | 13.9/13.9/4.2 | 18.4 |
| 12b | 2.16 | ddd | 13.9/13.9/4.6 | 18.4 |
| 13 | - | - | - | 125.0 |
| 14 | 6.27 | brs | - | 111.0 |
| 15 | 7.37 | brs | - | 143.1 |
| 16 | 7.22 | brs | - | 138.6 |
| 17 | 1.07 | d | 7.0 | 12.1 |
| 18 | - | - | - | 170.2 |
| 19a | 3.92 | brd | 7.6 | 73.0 |
| 19b | 5.31 | d | 7.6 | 73.0 |
| 20 | 0.87 | s | - | 19.38 |
15
14
16
13
11
20
1
17
8
2
10
5
7
3
19
18
700 MHz in CDCl3
*overlapped signals J unresolved
The chemical shifts were in agreement with the published data (Ceñal, 1997).

## Slide 5
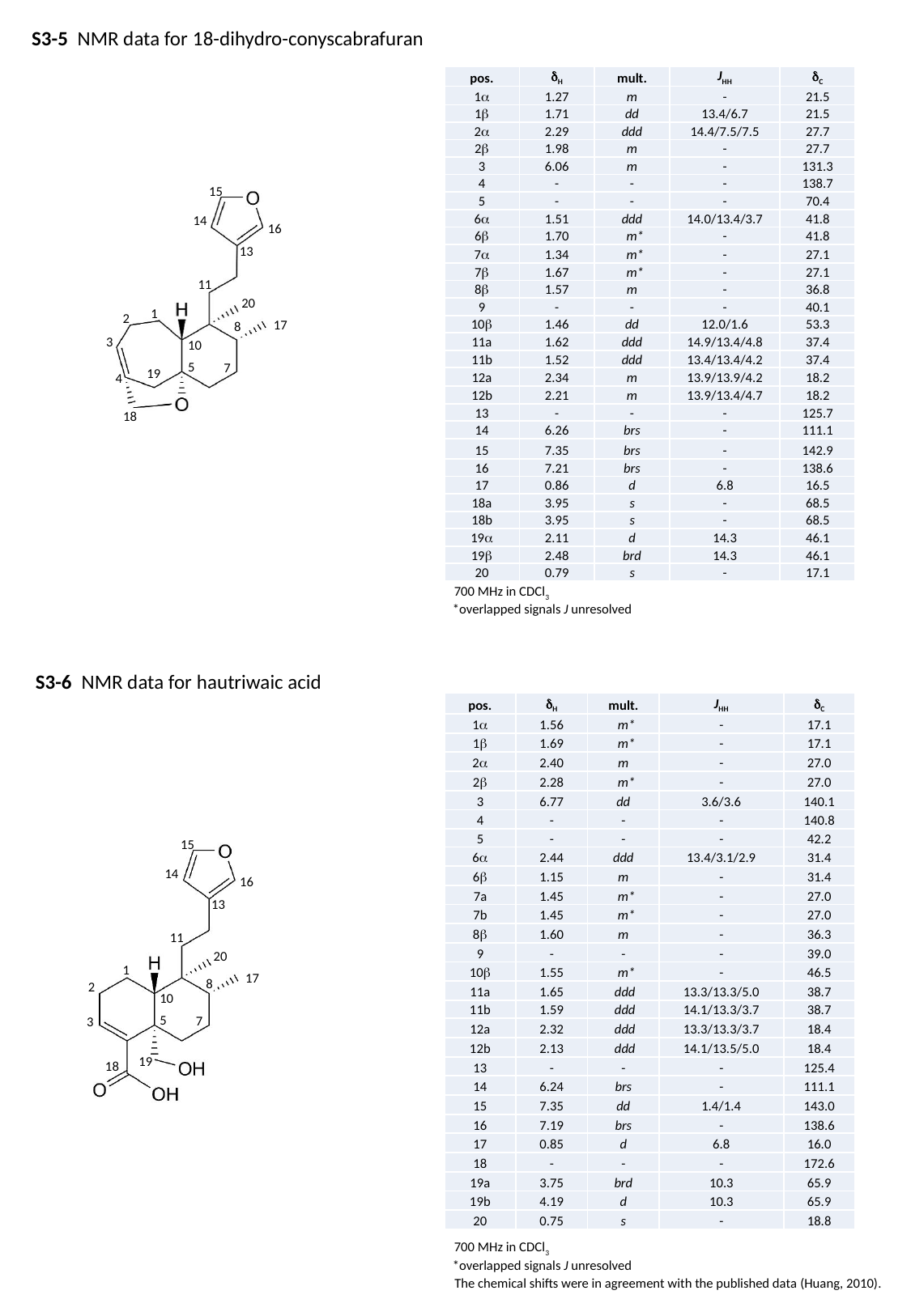

S3-5 NMR data for 18-dihydro-conyscabrafuran
| pos. | dH | mult. | JHH | dC |
| --- | --- | --- | --- | --- |
| 1a | 1.27 | m | - | 21.5 |
| 1b | 1.71 | dd | 13.4/6.7 | 21.5 |
| 2a | 2.29 | ddd | 14.4/7.5/7.5 | 27.7 |
| 2b | 1.98 | m | - | 27.7 |
| 3 | 6.06 | m | - | 131.3 |
| 4 | - | - | - | 138.7 |
| 5 | - | - | - | 70.4 |
| 6a | 1.51 | ddd | 14.0/13.4/3.7 | 41.8 |
| 6b | 1.70 | m\* | - | 41.8 |
| 7a | 1.34 | m\* | - | 27.1 |
| 7b | 1.67 | m\* | - | 27.1 |
| 8b | 1.57 | m | - | 36.8 |
| 9 | - | - | - | 40.1 |
| 10b | 1.46 | dd | 12.0/1.6 | 53.3 |
| 11a | 1.62 | ddd | 14.9/13.4/4.8 | 37.4 |
| 11b | 1.52 | ddd | 13.4/13.4/4.2 | 37.4 |
| 12a | 2.34 | m | 13.9/13.9/4.2 | 18.2 |
| 12b | 2.21 | m | 13.9/13.4/4.7 | 18.2 |
| 13 | - | - | - | 125.7 |
| 14 | 6.26 | brs | - | 111.1 |
| 15 | 7.35 | brs | - | 142.9 |
| 16 | 7.21 | brs | - | 138.6 |
| 17 | 0.86 | d | 6.8 | 16.5 |
| 18a | 3.95 | s | - | 68.5 |
| 18b | 3.95 | s | - | 68.5 |
| 19a | 2.11 | d | 14.3 | 46.1 |
| 19b | 2.48 | brd | 14.3 | 46.1 |
| 20 | 0.79 | s | - | 17.1 |
15
14
16
13
11
20
1
2
17
8
3
10
5
7
19
4
18
700 MHz in CDCl3
*overlapped signals J unresolved
S3-6 NMR data for hautriwaic acid
| pos. | dH | mult. | JHH | dC |
| --- | --- | --- | --- | --- |
| 1a | 1.56 | m\* | - | 17.1 |
| 1b | 1.69 | m\* | - | 17.1 |
| 2a | 2.40 | m | - | 27.0 |
| 2b | 2.28 | m\* | - | 27.0 |
| 3 | 6.77 | dd | 3.6/3.6 | 140.1 |
| 4 | - | - | - | 140.8 |
| 5 | - | - | - | 42.2 |
| 6a | 2.44 | ddd | 13.4/3.1/2.9 | 31.4 |
| 6b | 1.15 | m | - | 31.4 |
| 7a | 1.45 | m\* | - | 27.0 |
| 7b | 1.45 | m\* | - | 27.0 |
| 8b | 1.60 | m | - | 36.3 |
| 9 | - | - | - | 39.0 |
| 10b | 1.55 | m\* | - | 46.5 |
| 11a | 1.65 | ddd | 13.3/13.3/5.0 | 38.7 |
| 11b | 1.59 | ddd | 14.1/13.3/3.7 | 38.7 |
| 12a | 2.32 | ddd | 13.3/13.3/3.7 | 18.4 |
| 12b | 2.13 | ddd | 14.1/13.5/5.0 | 18.4 |
| 13 | - | - | - | 125.4 |
| 14 | 6.24 | brs | - | 111.1 |
| 15 | 7.35 | dd | 1.4/1.4 | 143.0 |
| 16 | 7.19 | brs | - | 138.6 |
| 17 | 0.85 | d | 6.8 | 16.0 |
| 18 | - | - | - | 172.6 |
| 19a | 3.75 | brd | 10.3 | 65.9 |
| 19b | 4.19 | d | 10.3 | 65.9 |
| 20 | 0.75 | s | - | 18.8 |
15
14
16
13
11
20
1
17
8
2
10
5
7
3
19
18
700 MHz in CDCl3
*overlapped signals J unresolved
The chemical shifts were in agreement with the published data (Huang, 2010).

## Slide 6
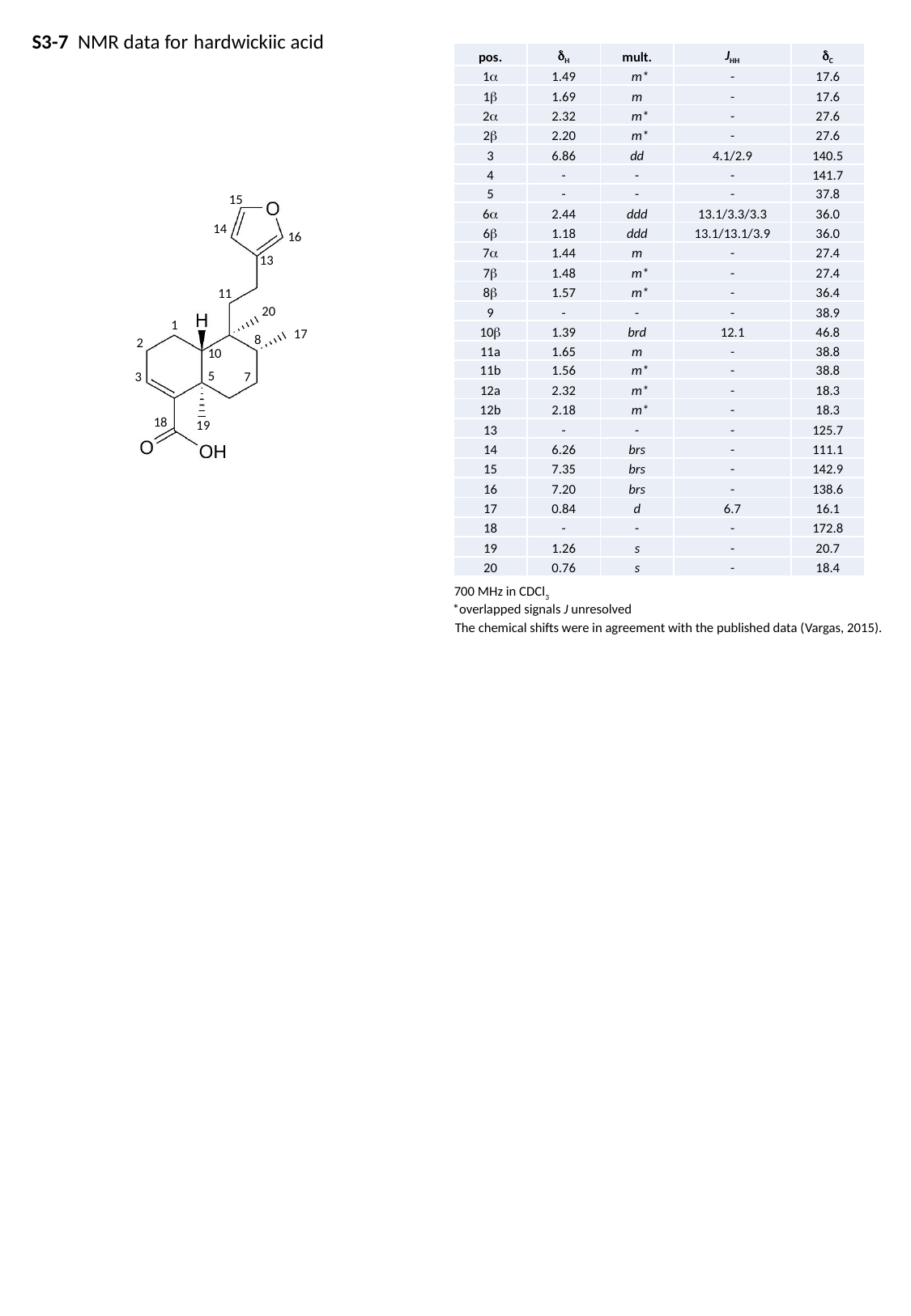

S3-7 NMR data for hardwickiic acid
| pos. | dH | mult. | JHH | dC |
| --- | --- | --- | --- | --- |
| 1a | 1.49 | m\* | - | 17.6 |
| 1b | 1.69 | m | - | 17.6 |
| 2a | 2.32 | m\* | - | 27.6 |
| 2b | 2.20 | m\* | - | 27.6 |
| 3 | 6.86 | dd | 4.1/2.9 | 140.5 |
| 4 | - | - | - | 141.7 |
| 5 | - | - | - | 37.8 |
| 6a | 2.44 | ddd | 13.1/3.3/3.3 | 36.0 |
| 6b | 1.18 | ddd | 13.1/13.1/3.9 | 36.0 |
| 7a | 1.44 | m | - | 27.4 |
| 7b | 1.48 | m\* | - | 27.4 |
| 8b | 1.57 | m\* | - | 36.4 |
| 9 | - | - | - | 38.9 |
| 10b | 1.39 | brd | 12.1 | 46.8 |
| 11a | 1.65 | m | - | 38.8 |
| 11b | 1.56 | m\* | - | 38.8 |
| 12a | 2.32 | m\* | - | 18.3 |
| 12b | 2.18 | m\* | - | 18.3 |
| 13 | - | - | - | 125.7 |
| 14 | 6.26 | brs | - | 111.1 |
| 15 | 7.35 | brs | - | 142.9 |
| 16 | 7.20 | brs | - | 138.6 |
| 17 | 0.84 | d | 6.7 | 16.1 |
| 18 | - | - | - | 172.8 |
| 19 | 1.26 | s | - | 20.7 |
| 20 | 0.76 | s | - | 18.4 |
15
14
16
13
11
20
1
17
8
2
10
5
7
3
18
19
700 MHz in CDCl3
*overlapped signals J unresolved
The chemical shifts were in agreement with the published data (Vargas, 2015).

## Slide 7
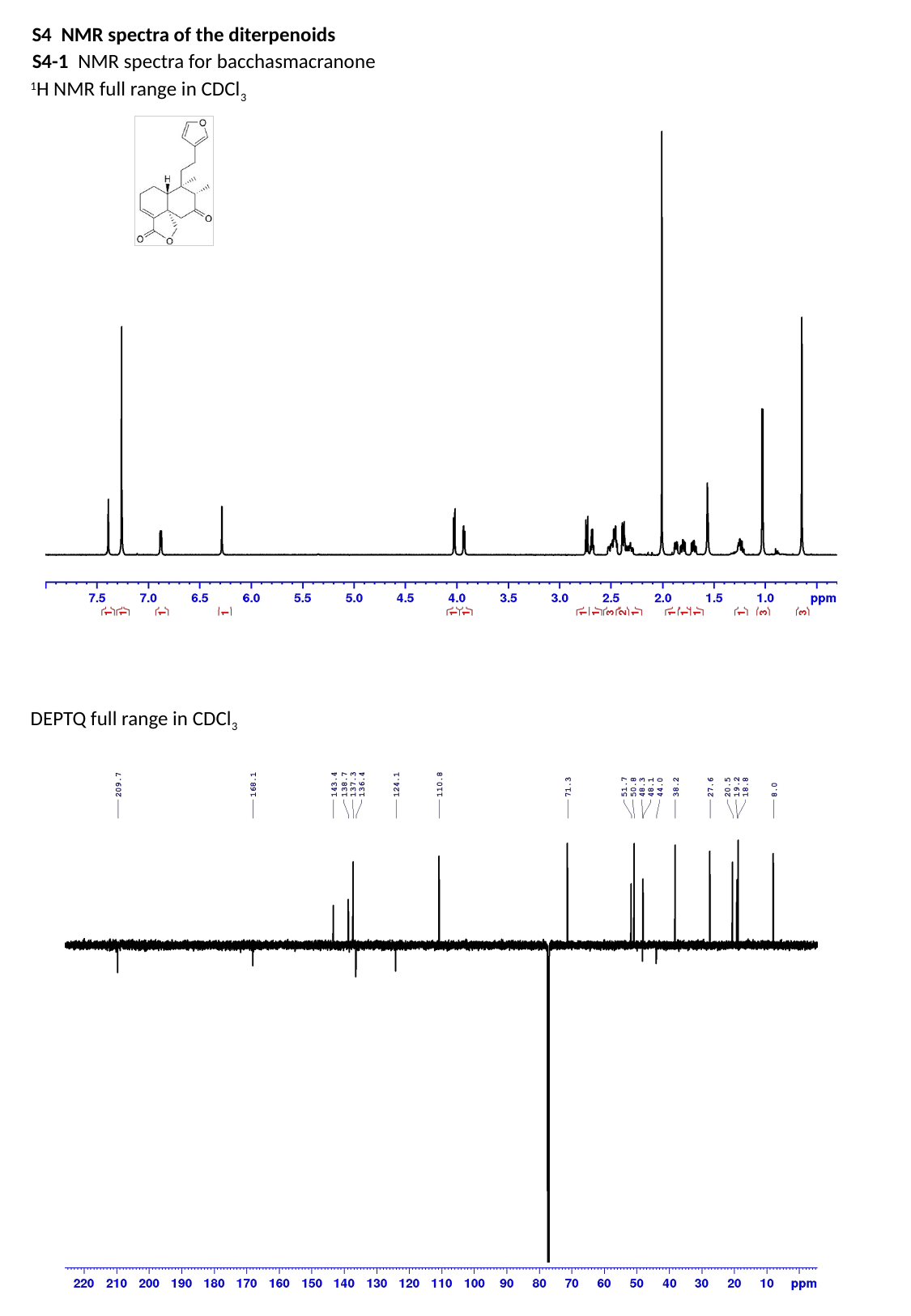

S4 NMR spectra of the diterpenoids
S4-1 NMR spectra for bacchasmacranone
1H NMR full range in CDCl3
DEPTQ full range in CDCl3

## Slide 8
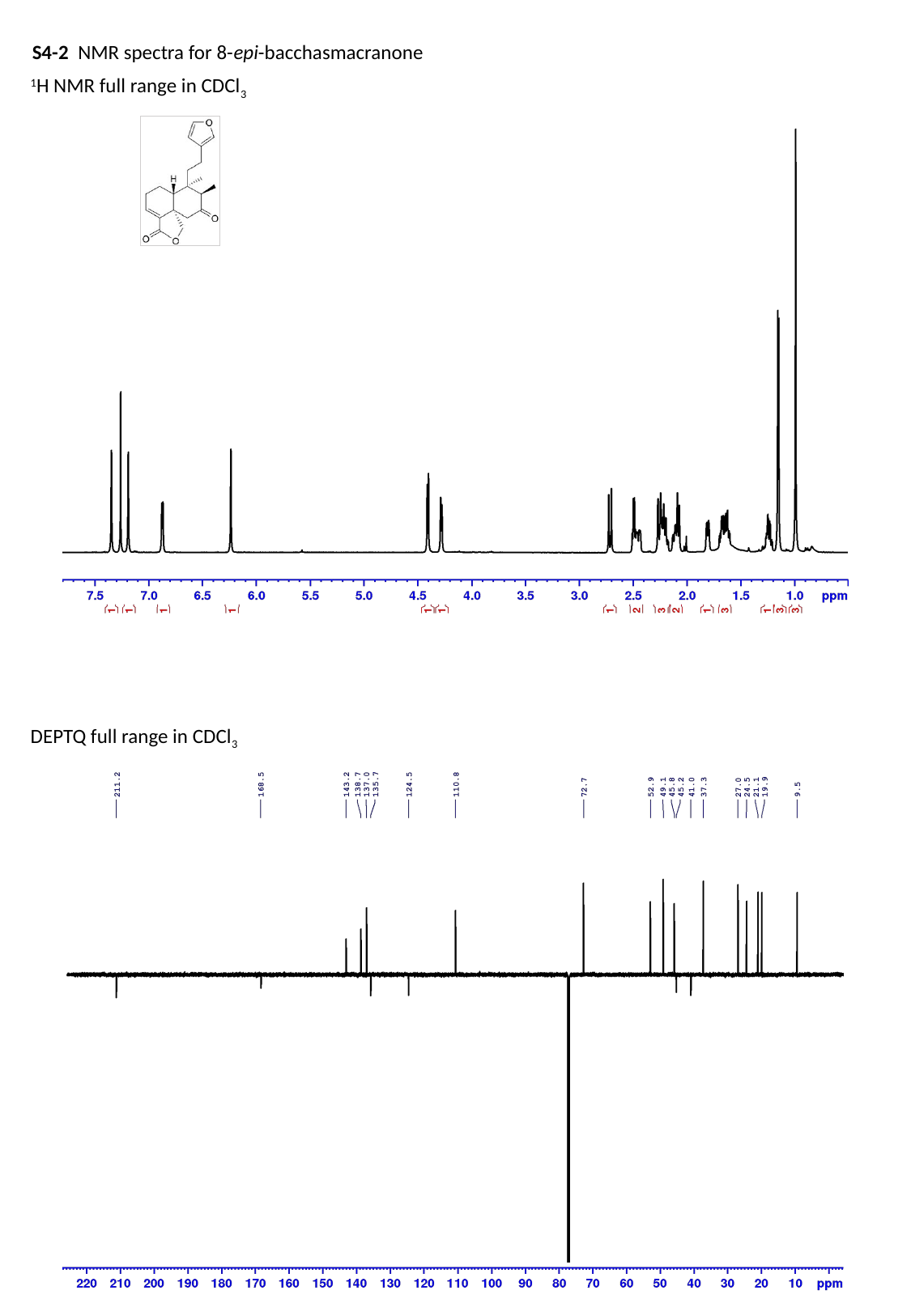

S4-2 NMR spectra for 8-epi-bacchasmacranone
1H NMR full range in CDCl3
DEPTQ full range in CDCl3

## Slide 9
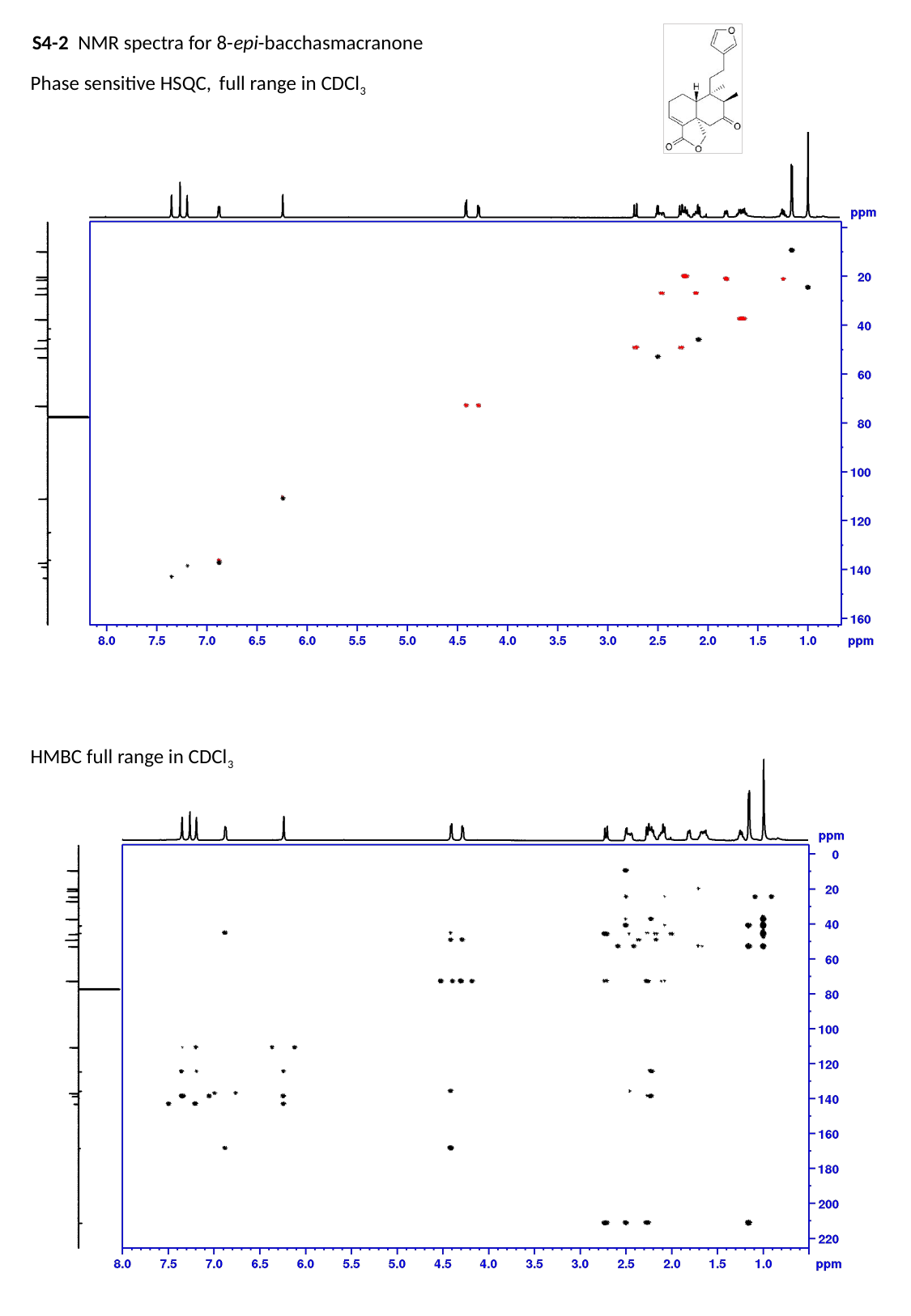

S4-2 NMR spectra for 8-epi-bacchasmacranone
Phase sensitive HSQC, full range in CDCl3
HMBC full range in CDCl3

## Slide 10
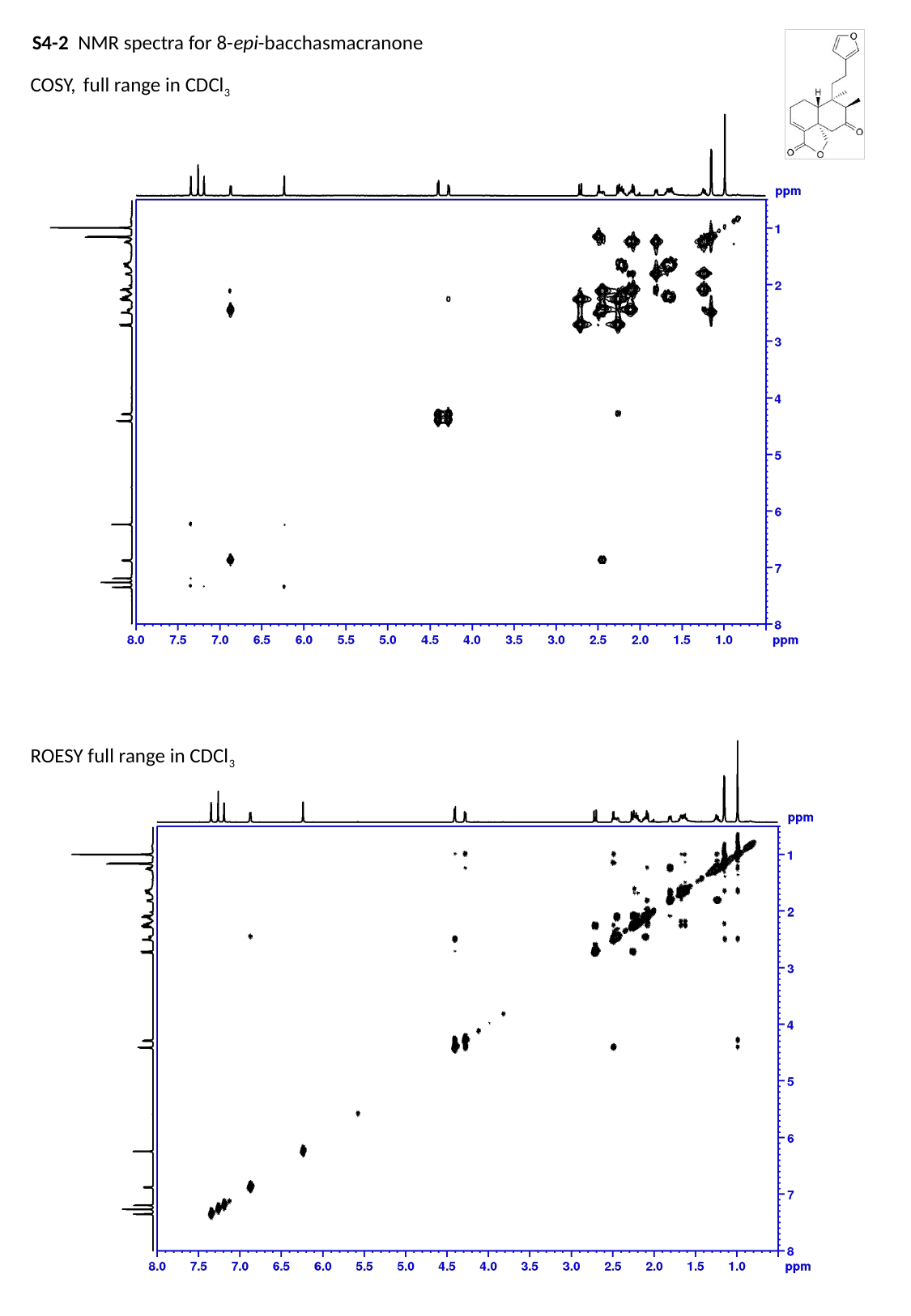

S4-2 NMR spectra for 8-epi-bacchasmacranone
COSY, full range in CDCl3
ROESY full range in CDCl3

## Slide 11
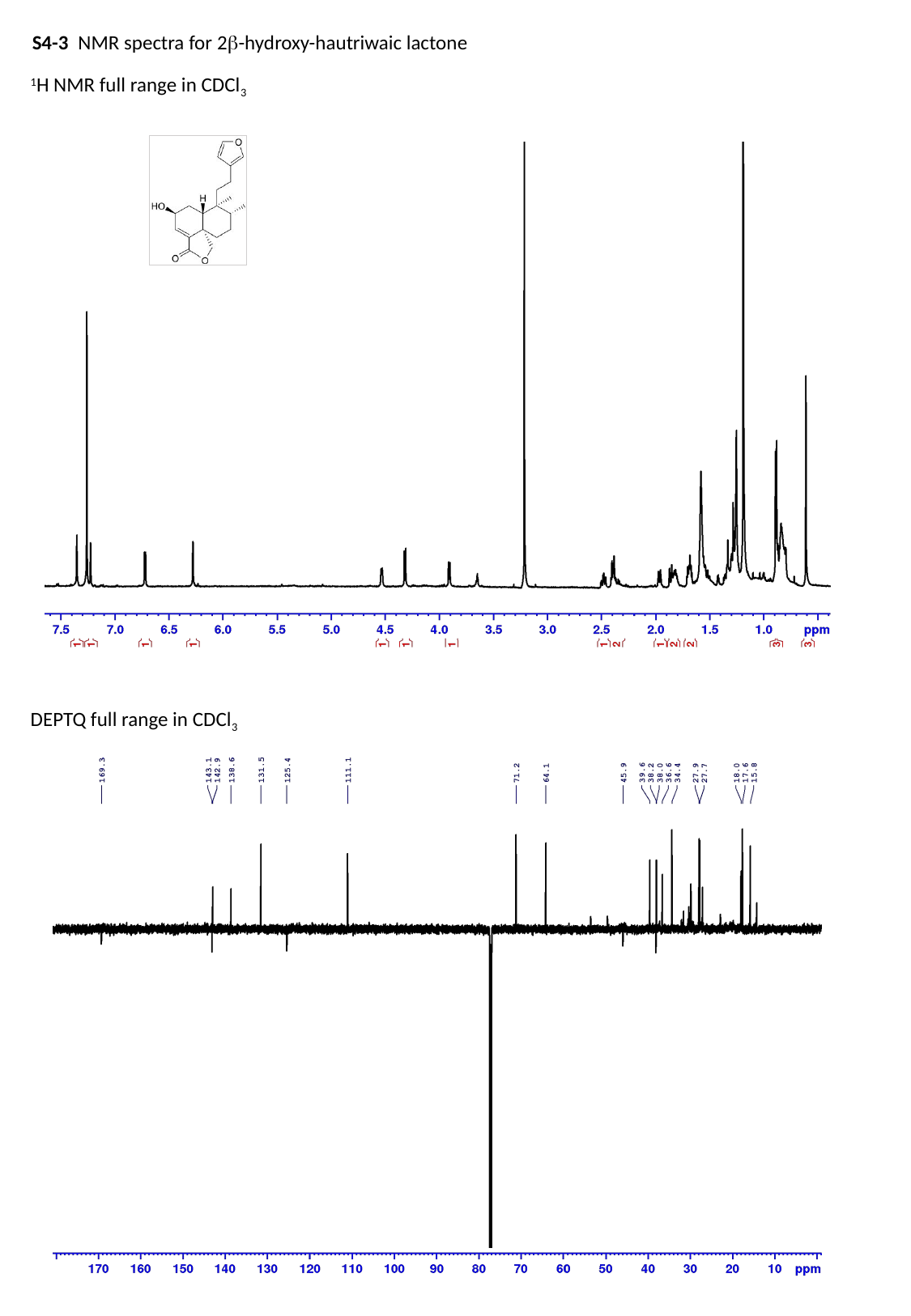

S4-3 NMR spectra for 2b-hydroxy-hautriwaic lactone
1H NMR full range in CDCl3
DEPTQ full range in CDCl3

## Slide 12
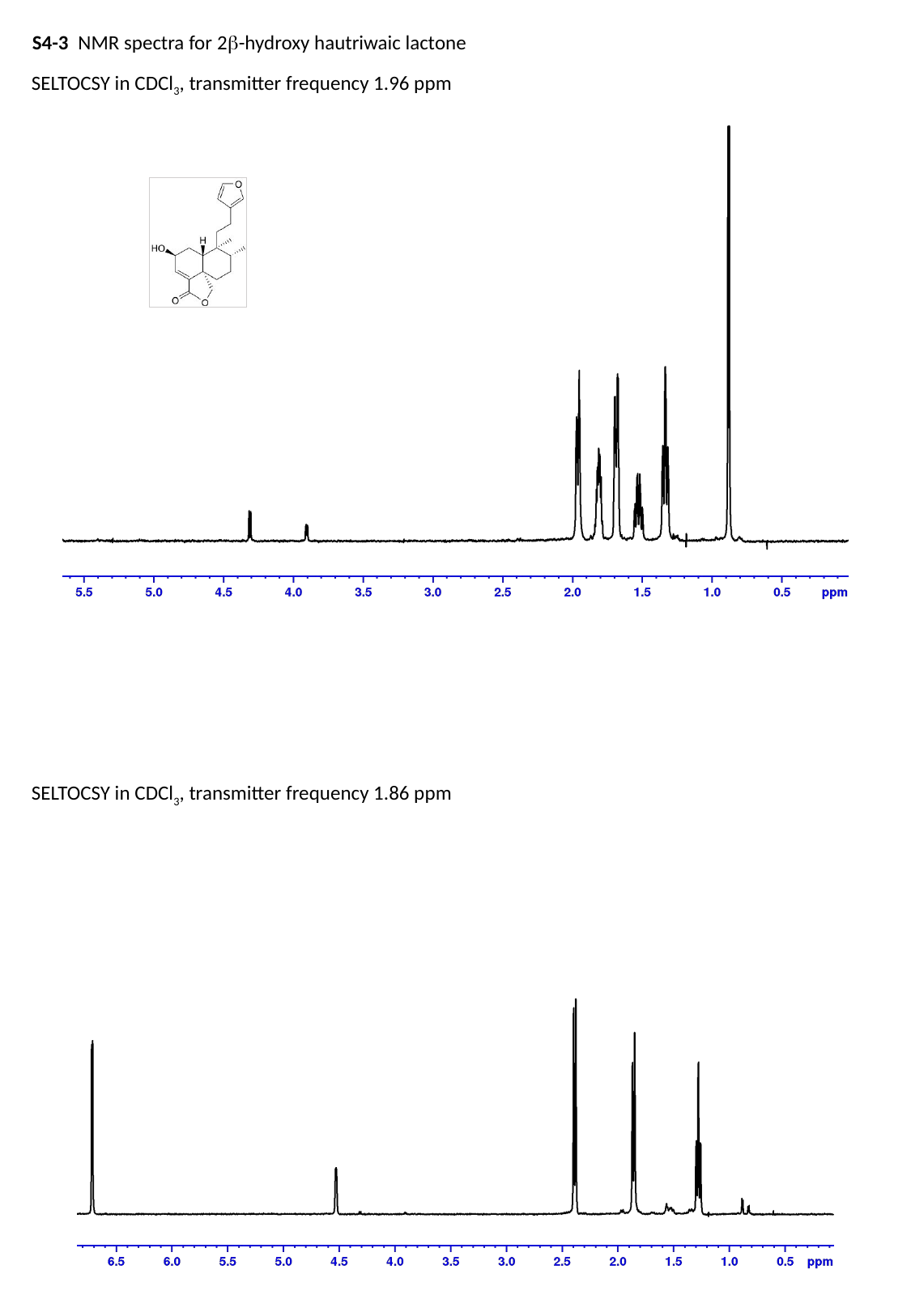

S4-3 NMR spectra for 2b-hydroxy hautriwaic lactone
SELTOCSY in CDCl3, transmitter frequency 1.96 ppm
SELTOCSY in CDCl3, transmitter frequency 1.86 ppm

## Slide 13
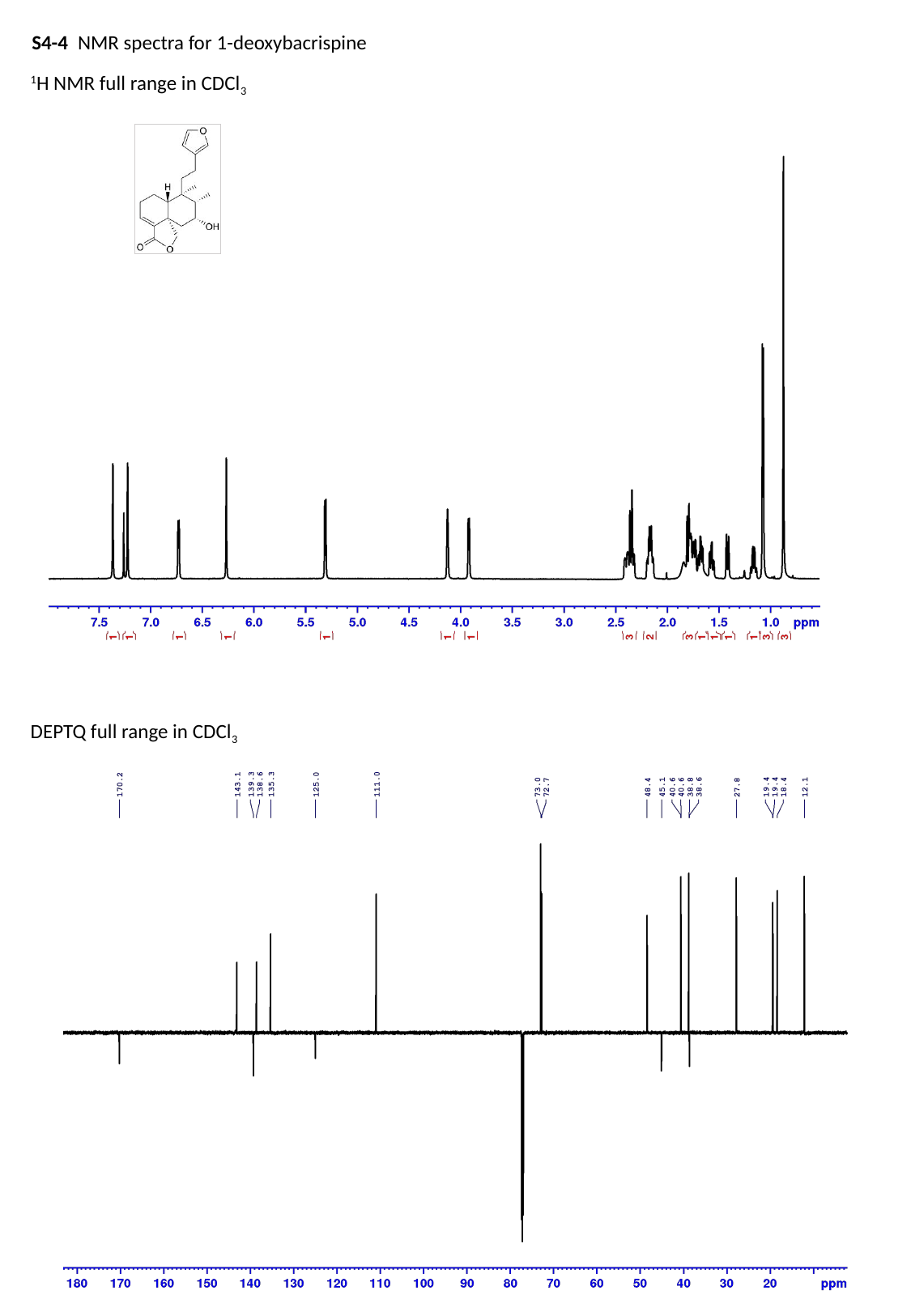

S4-4 NMR spectra for 1-deoxybacrispine
1H NMR full range in CDCl3
DEPTQ full range in CDCl3

## Slide 14
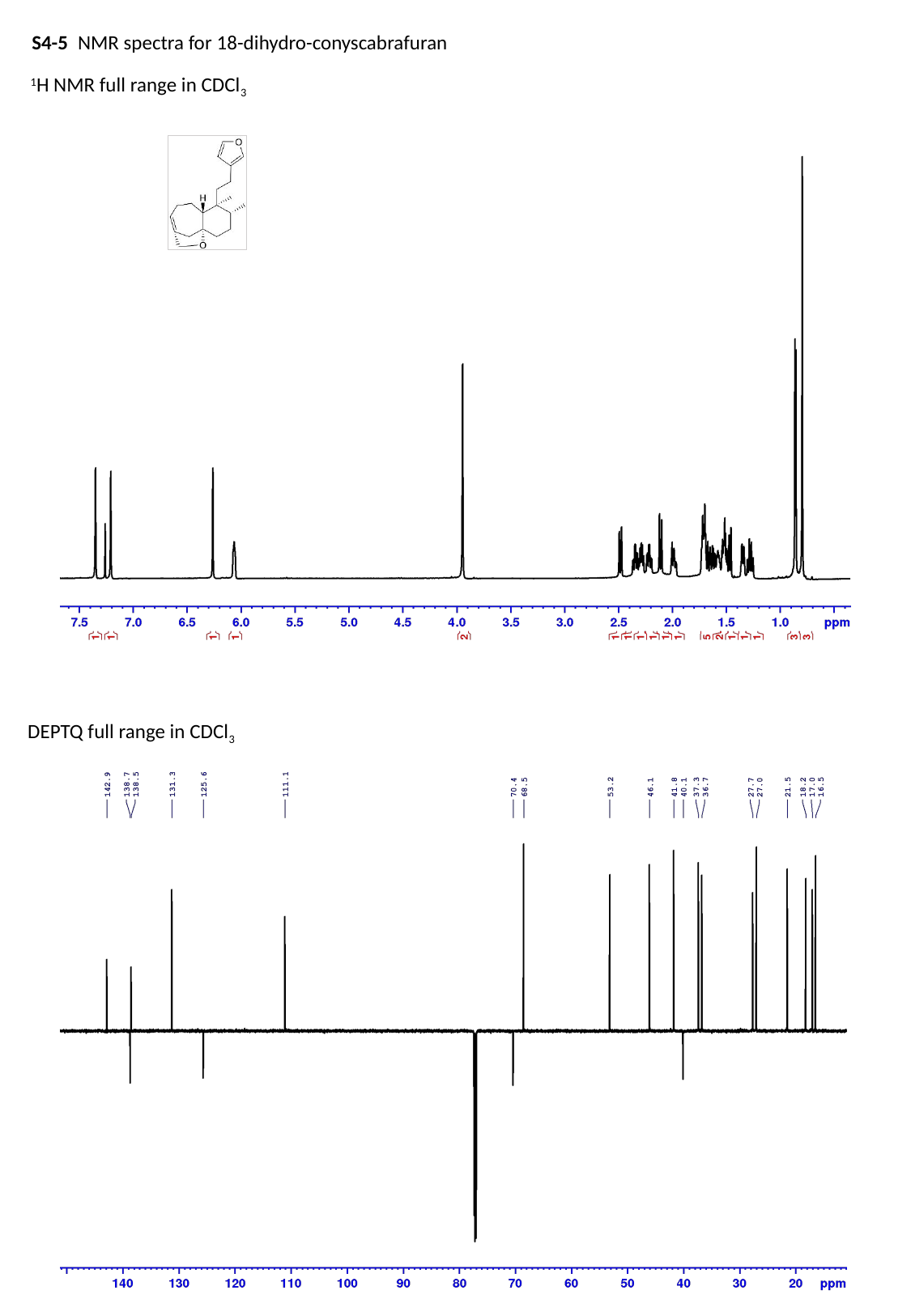

S4-5 NMR spectra for 18-dihydro-conyscabrafuran
1H NMR full range in CDCl3
DEPTQ full range in CDCl3

## Slide 15
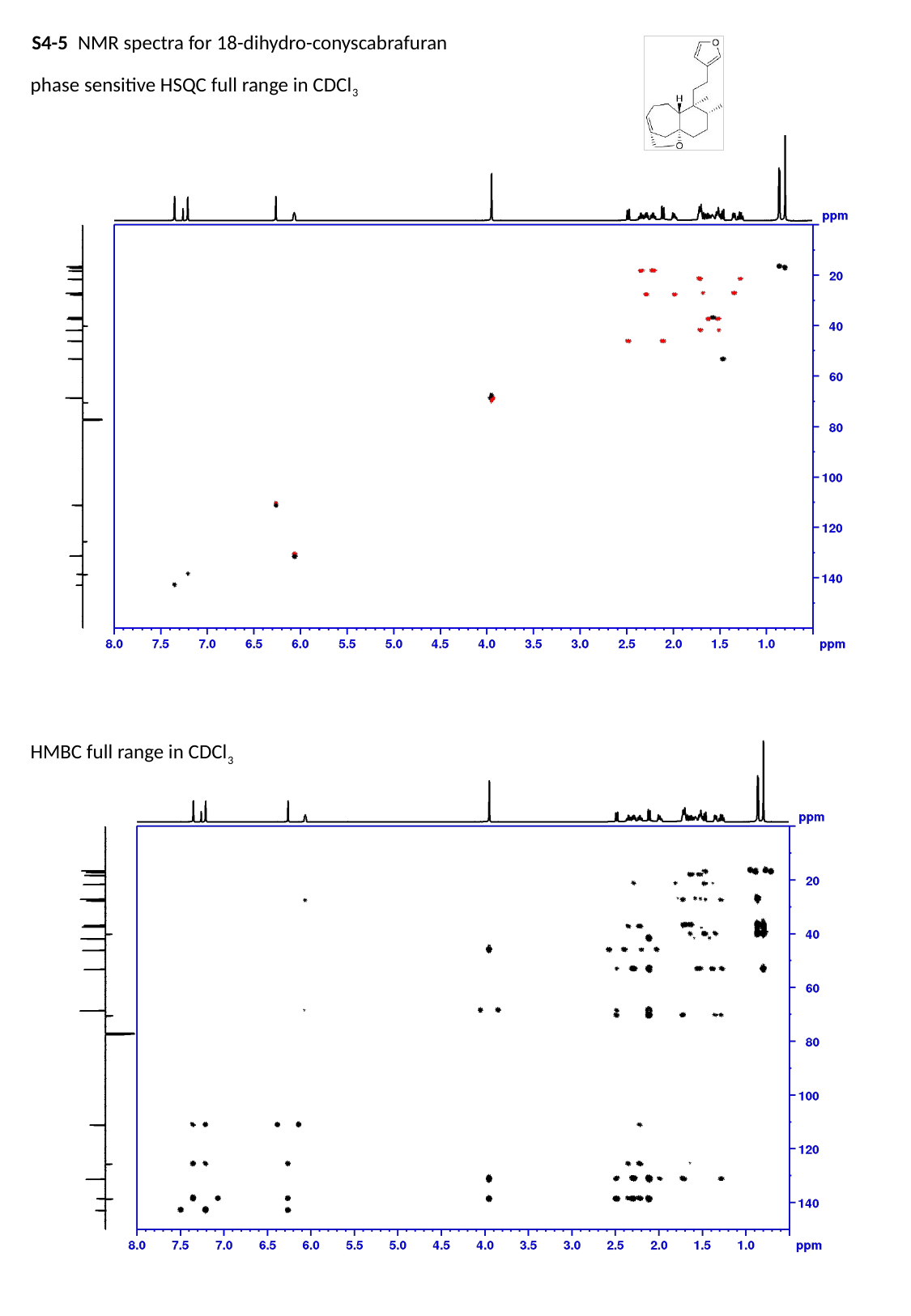

S4-5 NMR spectra for 18-dihydro-conyscabrafuran
phase sensitive HSQC full range in CDCl3
HMBC full range in CDCl3

## Slide 16
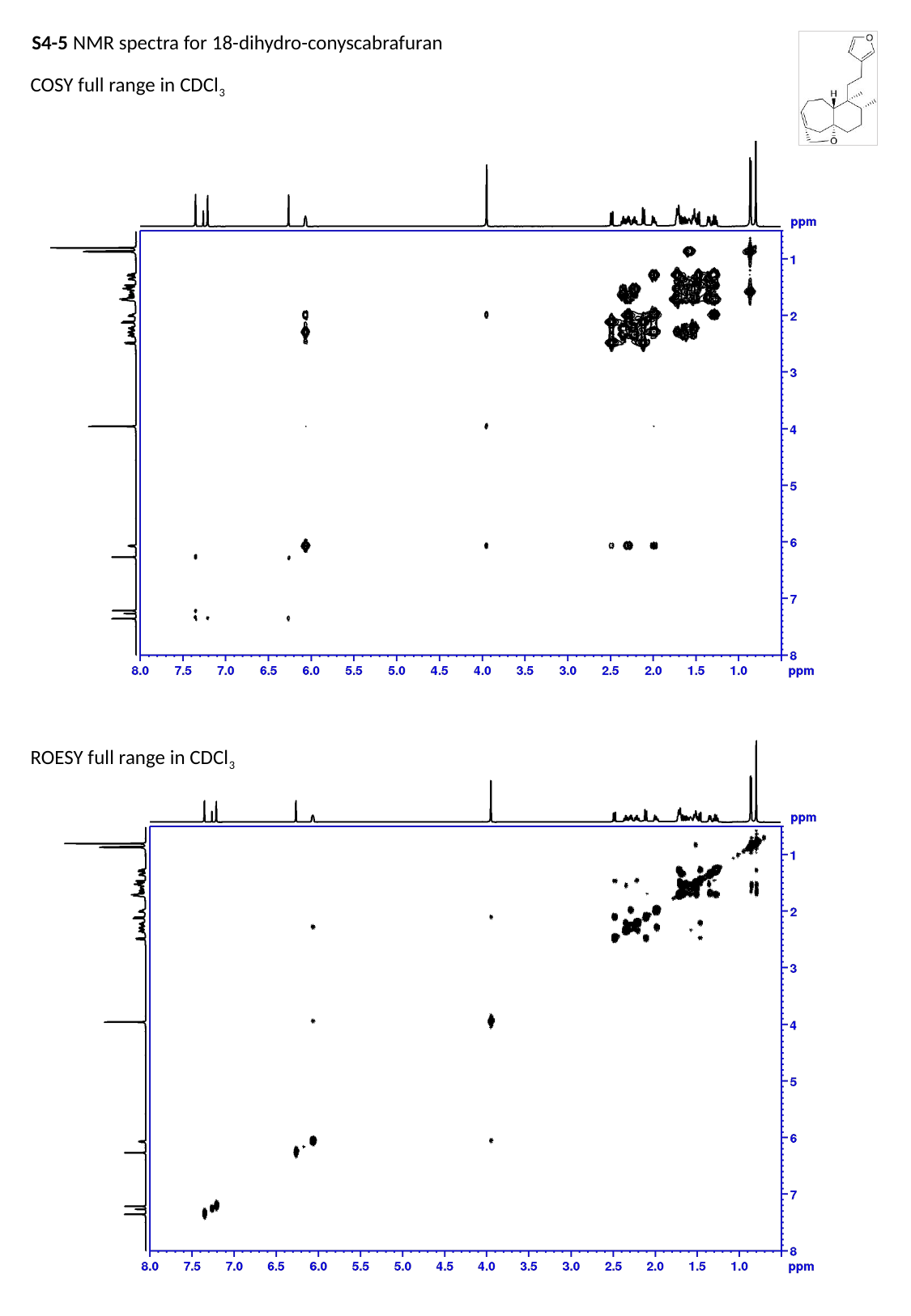

S4-5 NMR spectra for 18-dihydro-conyscabrafuran
COSY full range in CDCl3
ROESY full range in CDCl3

## Slide 17
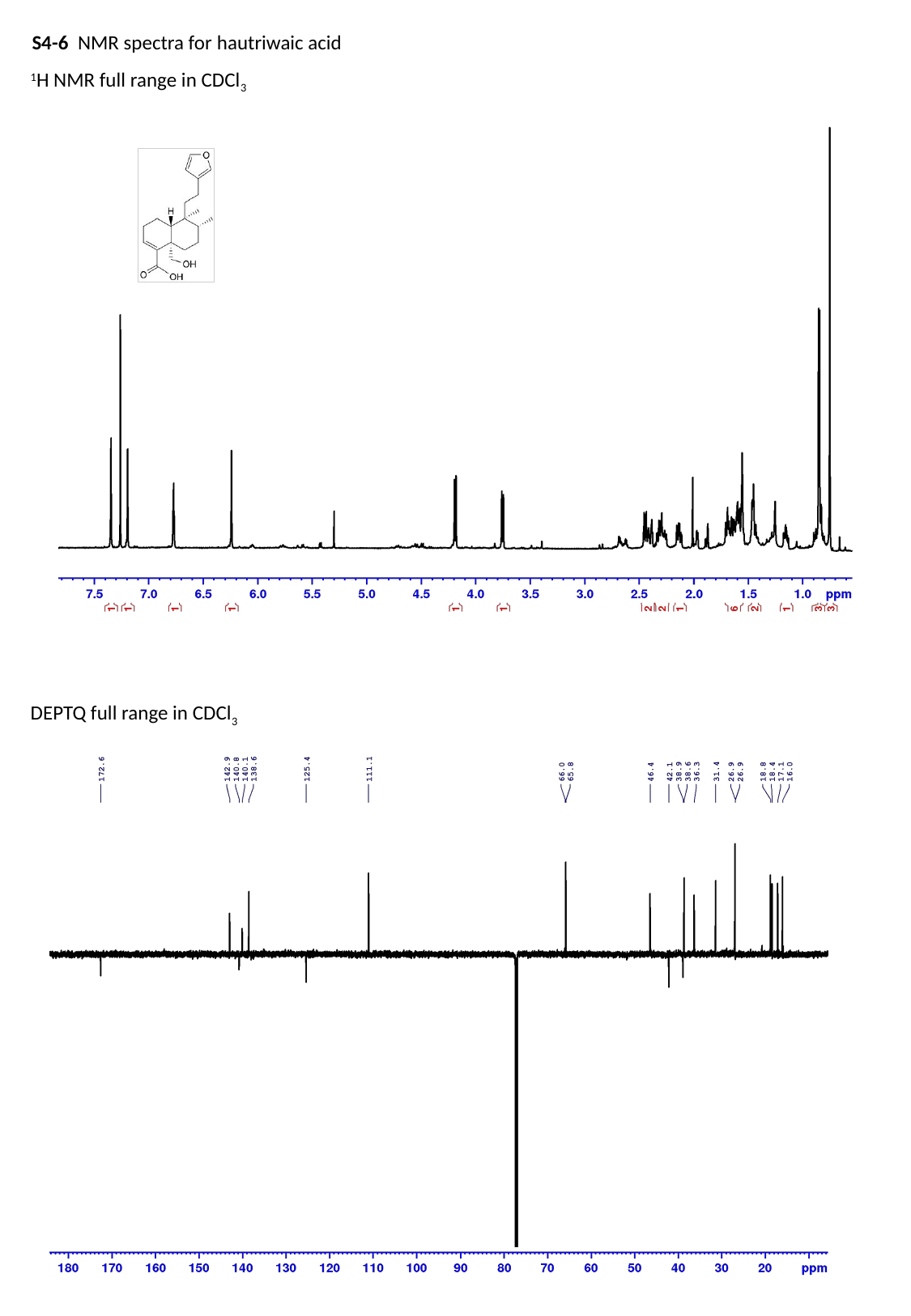

S4-6 NMR spectra for hautriwaic acid
1H NMR full range in CDCl3
DEPTQ full range in CDCl3

## Slide 18
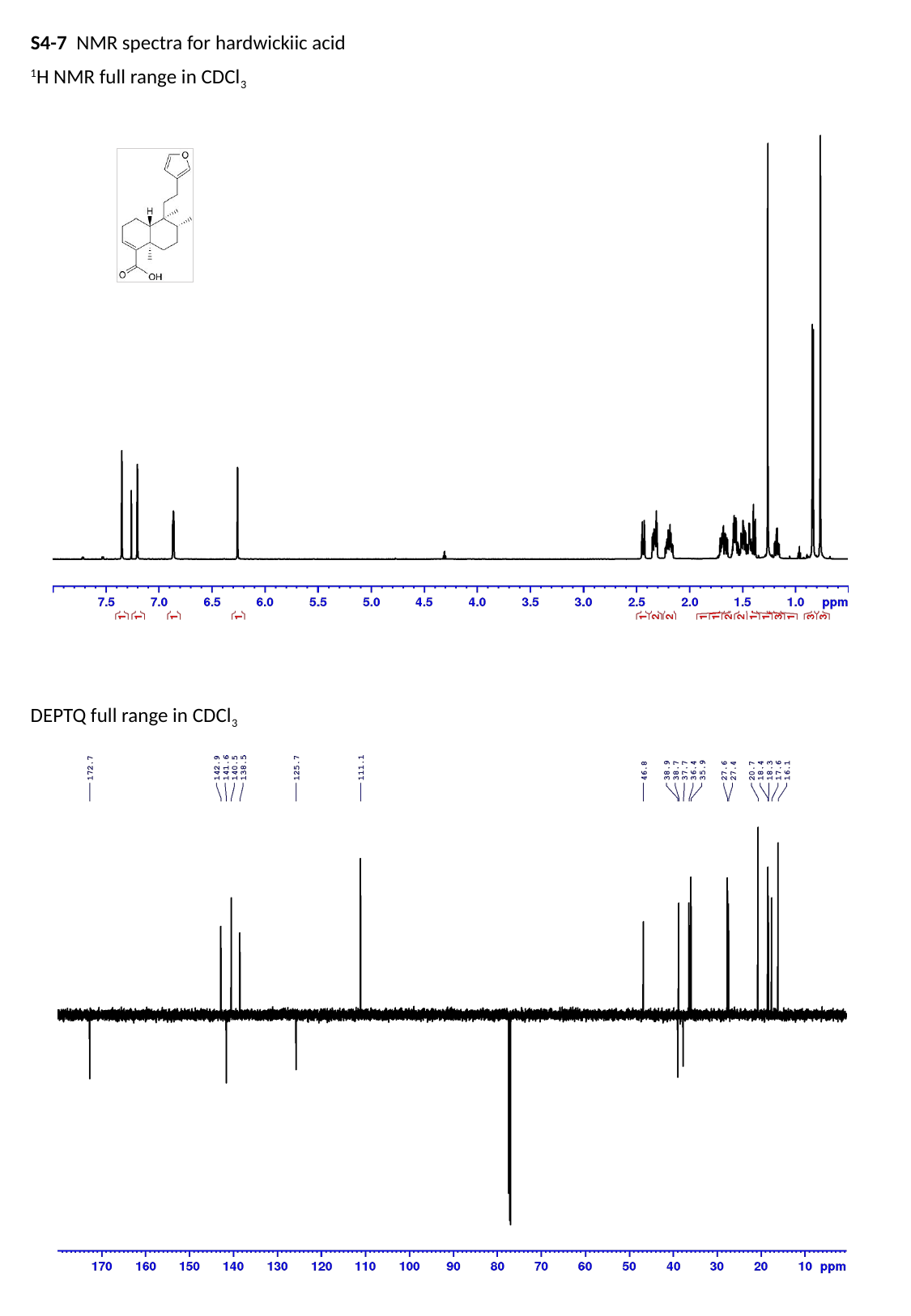

S4-7 NMR spectra for hardwickiic acid
1H NMR full range in CDCl3
DEPTQ full range in CDCl3
