# Supplementary material for: Consequences of interspecific plant hybridization on metabolic diversity in naturally occurring hybrid swarms
Source: Plant J. 2025 Aug 26;123(4):e70444. doi: 10.1111/tpj.70444 (PMC12380477; doi:10.1111/tpj.70444)
Supplement: Supplementary file 6 — Figure S1. GBS results. Group: all samples, reads were mapped against the reference created in (Schneider & Hellwig, 2024). Figure S2. Principal component analysis based on the profiles of semi‐polar metabolites, obtained by LC‐MS measurements, for the samples morphologically characterized as B. macraei, B. linearis, and B. × intermedia. Figure S3. Genetic cluster assignments (A, K = 3; B, K = 6) of the investigated Baccharis taxa using LEA v. 2.6.0 shown as barplots. Figure S4. Examples of molecular networks containing features abundant in either (A) B. macraei and B. × intermedia or (B) B. linearis and B. × intermedia. Each node corresponds to a metabolic feature, edges present when the cosine similarity between spectra is >0.7. Figure S5. Molecular network containing compounds annotated as flavonoids. Each node corresponds to a metabolic feature, edges present when the cosine similarity between spectra is >0.7. Yellow, B. linearis; green, B. × intermedia; blue, B. macraei. Figure S6. Molecular network containing compounds annotated as diterpenoids. Each node corresponds to a metabolic feature, edges present when the cosine similarity between spectra is >0.7. Yellow, B. linearis; green, B. × intermedia; blue, B. macraei. Black circles indicate a high abundance. Figure S7. Molecular network containing compounds annotated as triterpenoids. Each node corresponds to a metabolic feature, edges present when the cosine similarity between spectra is >0.7. Yellow, B. linearis; green, B. × intermedia; blue, B. macraei. Black circles indicate a high abundance. Figure S8. Pie‐charts summarizing the chemical superclass annotation of (A) metabolic features classified as primary metabolites and (B) metabolic features classified as secondary metabolites. The numbers represent the number of features in each chemical class. Figure S9. Principal component analysis based on the profiles of the metabolic features annotated as primary metabolites, colored according the location (ordere [file TPJ-123-0-s004.pptx]

## Slide 1
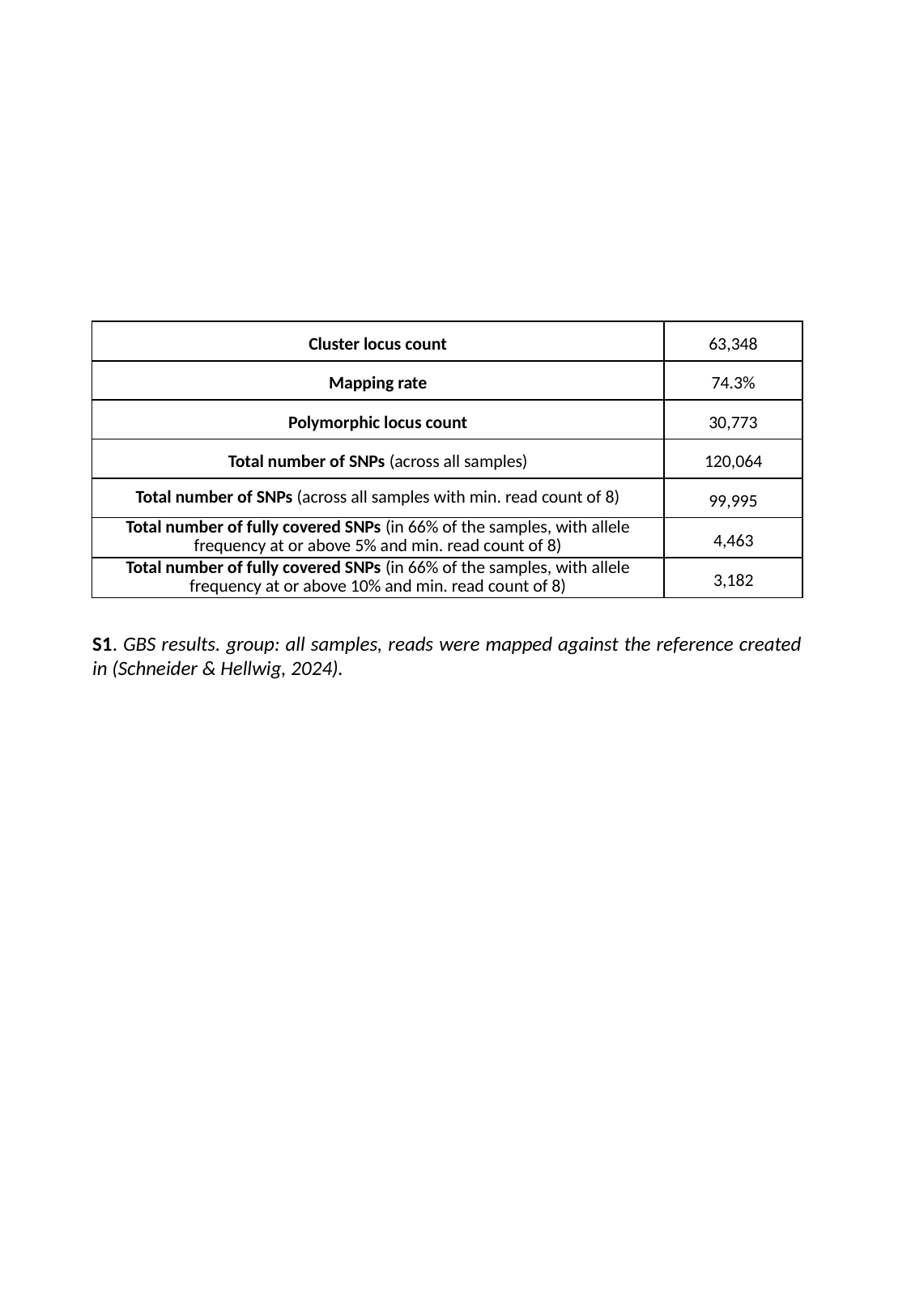

| Cluster locus count | 63,348 |
| --- | --- |
| Mapping rate | 74.3% |
| Polymorphic locus count | 30,773 |
| Total number of SNPs (across all samples) | 120,064 |
| Total number of SNPs (across all samples with min. read count of 8) | 99,995 |
| Total number of fully covered SNPs (in 66% of the samples, with allele frequency at or above 5% and min. read count of 8) | 4,463 |
| Total number of fully covered SNPs (in 66% of the samples, with allele frequency at or above 10% and min. read count of 8) | 3,182 |
S1. GBS results. group: all samples, reads were mapped against the reference created in (Schneider & Hellwig, 2024).

## Slide 2
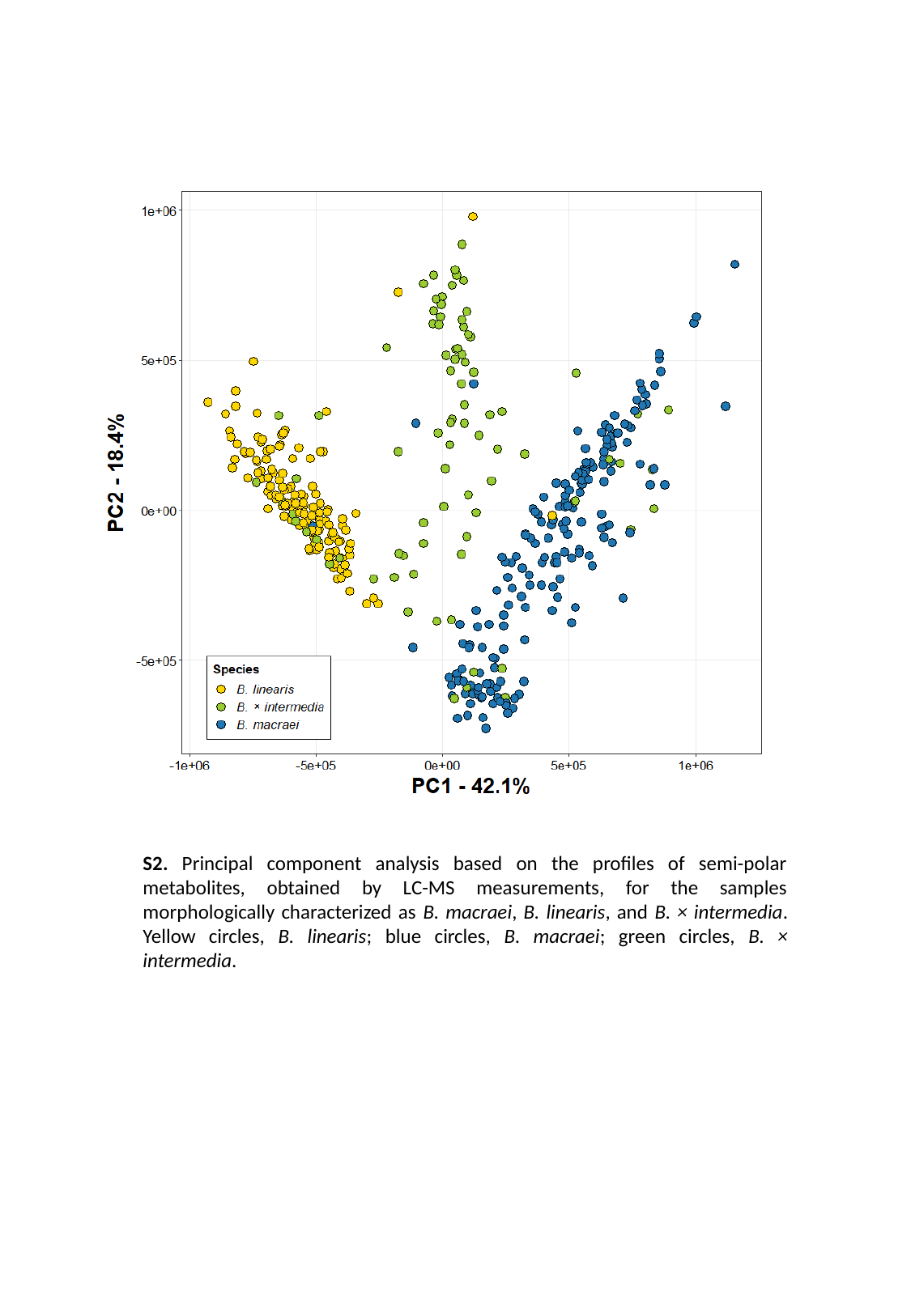

S2. Principal component analysis based on the profiles of semi-polar metabolites, obtained by LC-MS measurements, for the samples morphologically characterized as B. macraei, B. linearis, and B. × intermedia. Yellow circles, B. linearis; blue circles, B. macraei; green circles, B. × intermedia.

## Slide 3
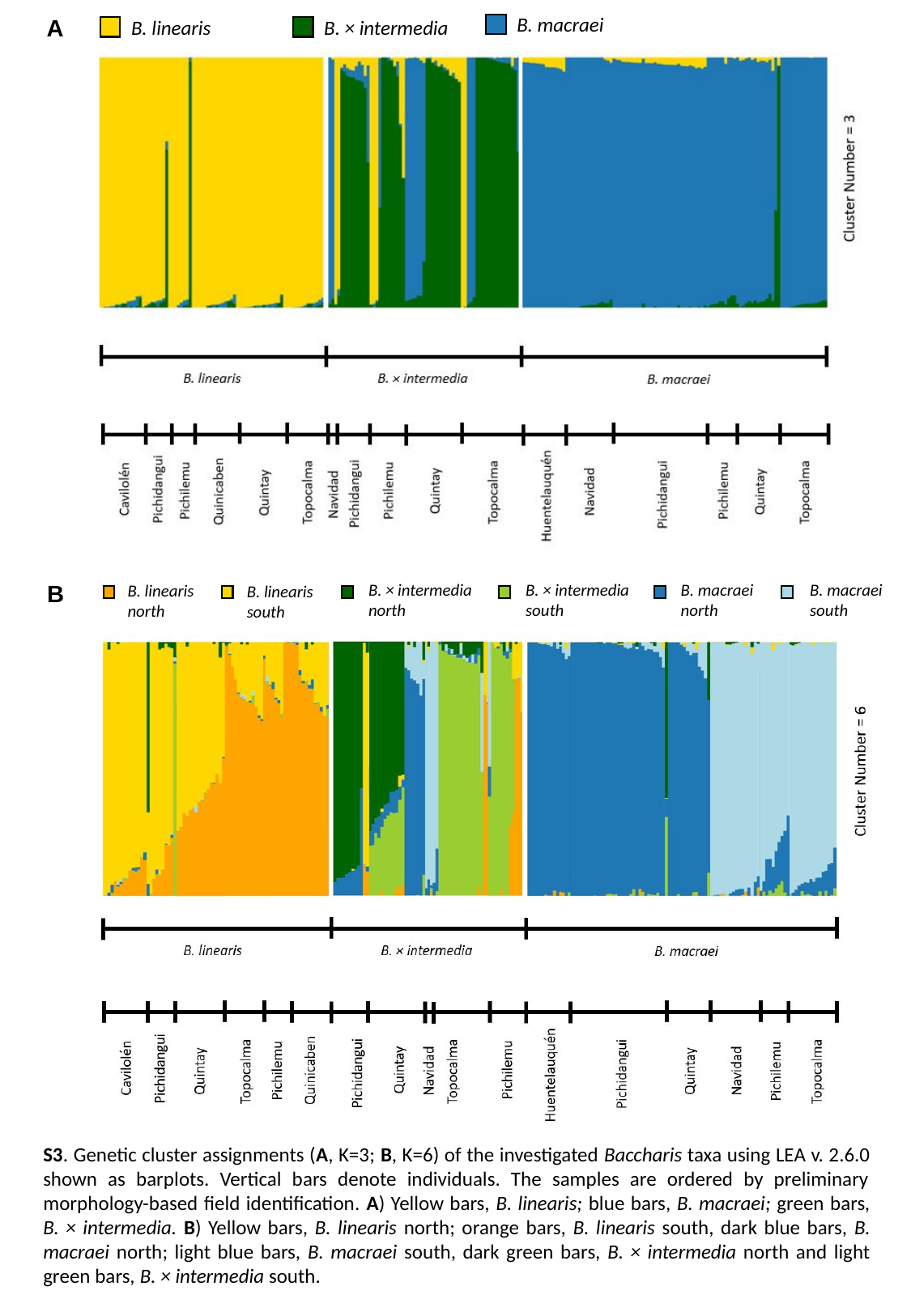

B. macraei
B. linearis
B. × intermedia
A
B
B. × intermedia north
B. × intermedia south
B. macraei north
B. macraei south
B. linearis north
B. linearis south
S3. Genetic cluster assignments (A, K=3; B, K=6) of the investigated Baccharis taxa using LEA v. 2.6.0 shown as barplots. Vertical bars denote individuals. The samples are ordered by preliminary morphology-based field identification. A) Yellow bars, B. linearis; blue bars, B. macraei; green bars, B. × intermedia. B) Yellow bars, B. linearis north; orange bars, B. linearis south, dark blue bars, B. macraei north; light blue bars, B. macraei south, dark green bars, B. × intermedia north and light green bars, B. × intermedia south.

## Slide 4
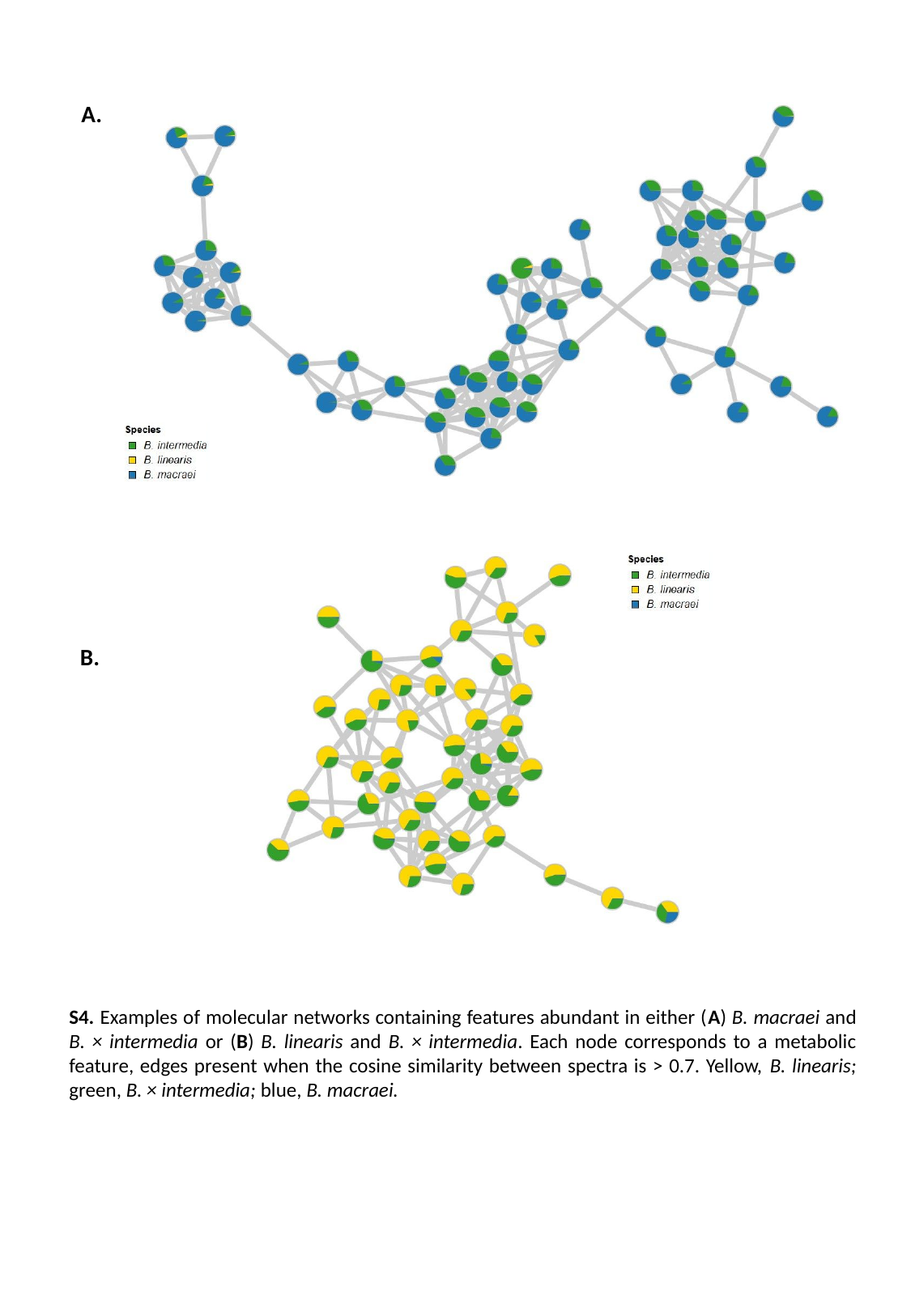

A.
B.
S4. Examples of molecular networks containing features abundant in either (A) B. macraei and B. × intermedia or (B) B. linearis and B. × intermedia. Each node corresponds to a metabolic feature, edges present when the cosine similarity between spectra is > 0.7. Yellow, B. linearis; green, B. × intermedia; blue, B. macraei.

## Slide 5
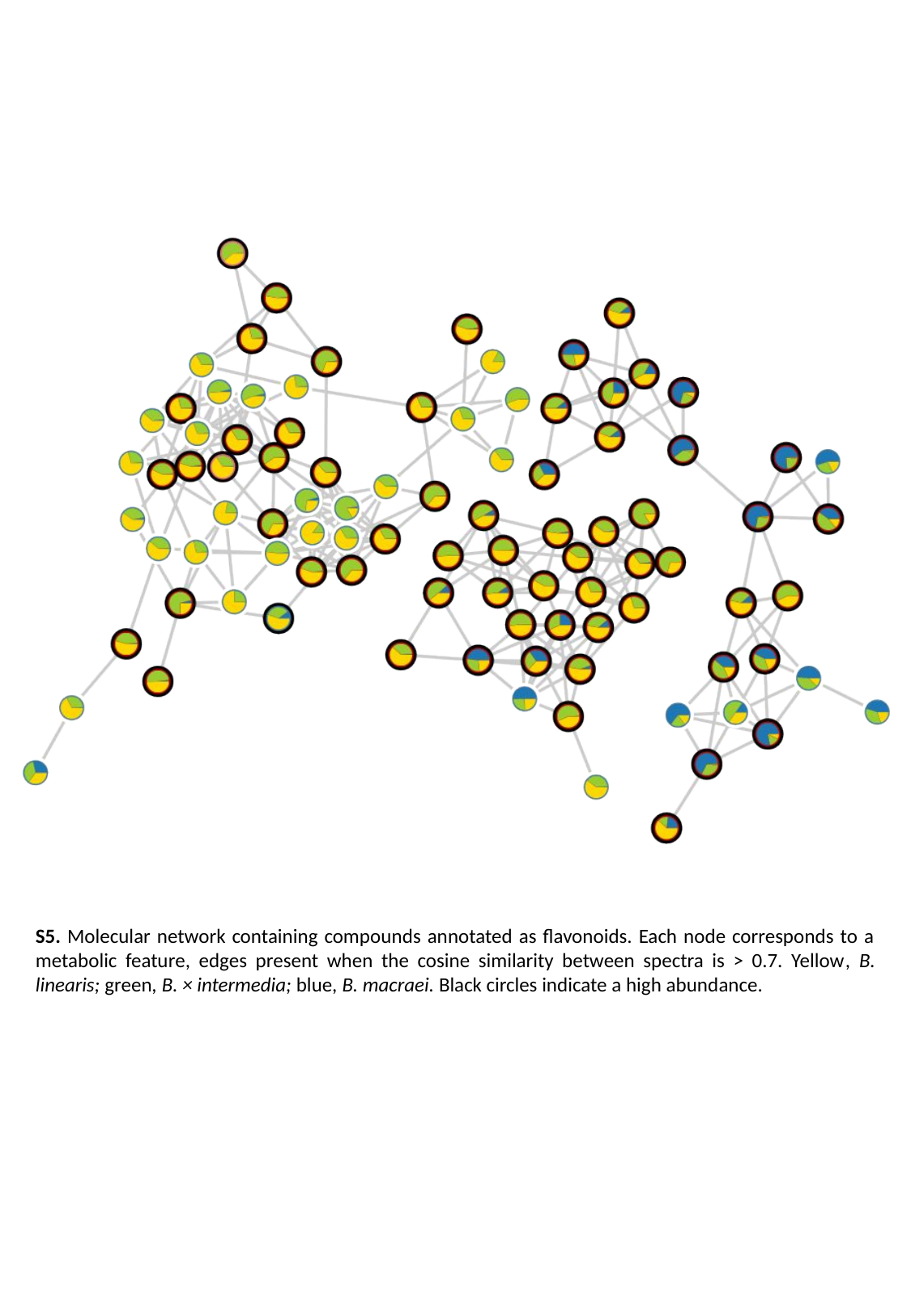

S5. Molecular network containing compounds annotated as flavonoids. Each node corresponds to a metabolic feature, edges present when the cosine similarity between spectra is > 0.7. Yellow, B. linearis; green, B. × intermedia; blue, B. macraei. Black circles indicate a high abundance.

## Slide 6
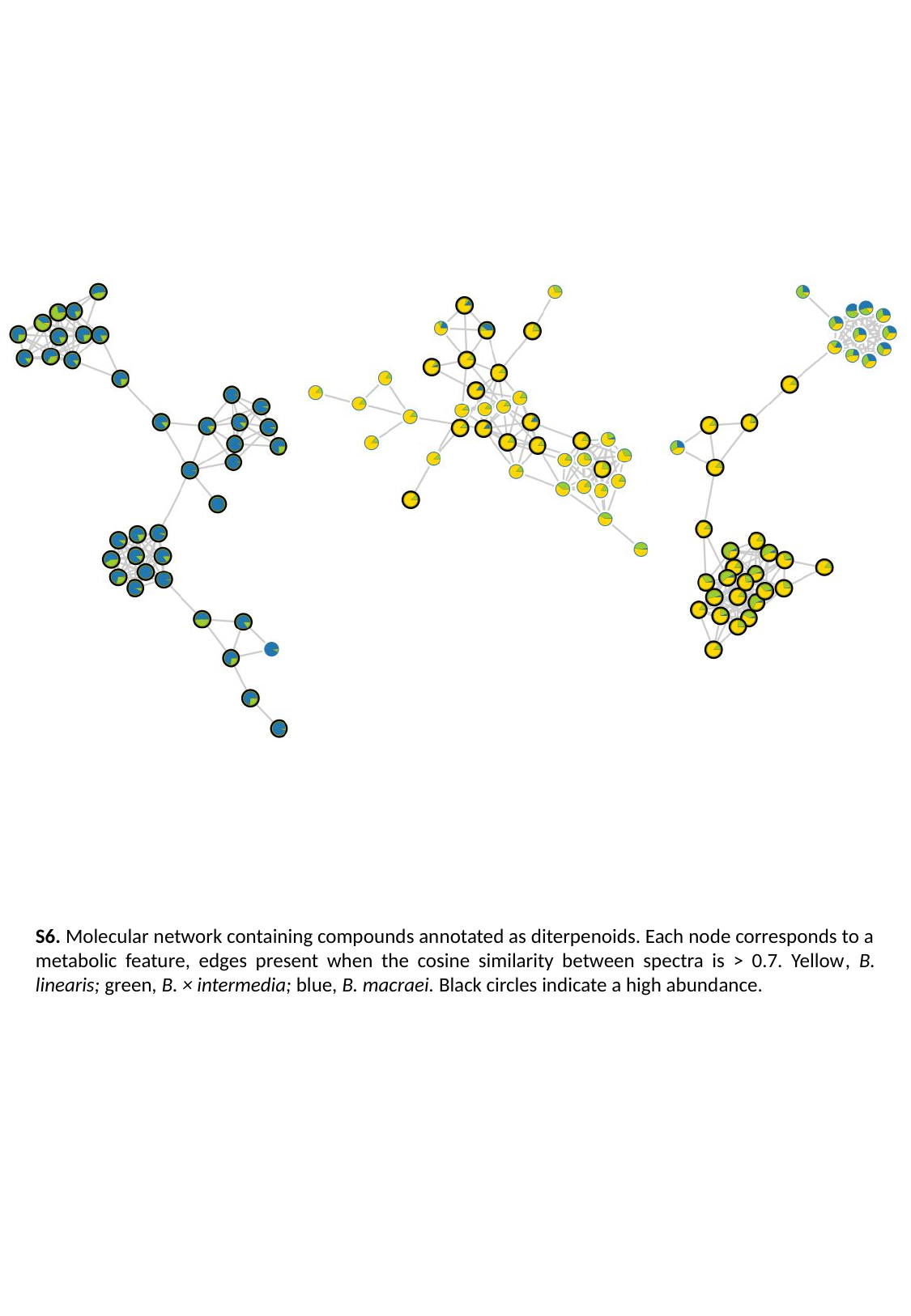

S6. Molecular network containing compounds annotated as diterpenoids. Each node corresponds to a metabolic feature, edges present when the cosine similarity between spectra is > 0.7. Yellow, B. linearis; green, B. × intermedia; blue, B. macraei. Black circles indicate a high abundance.

## Slide 7
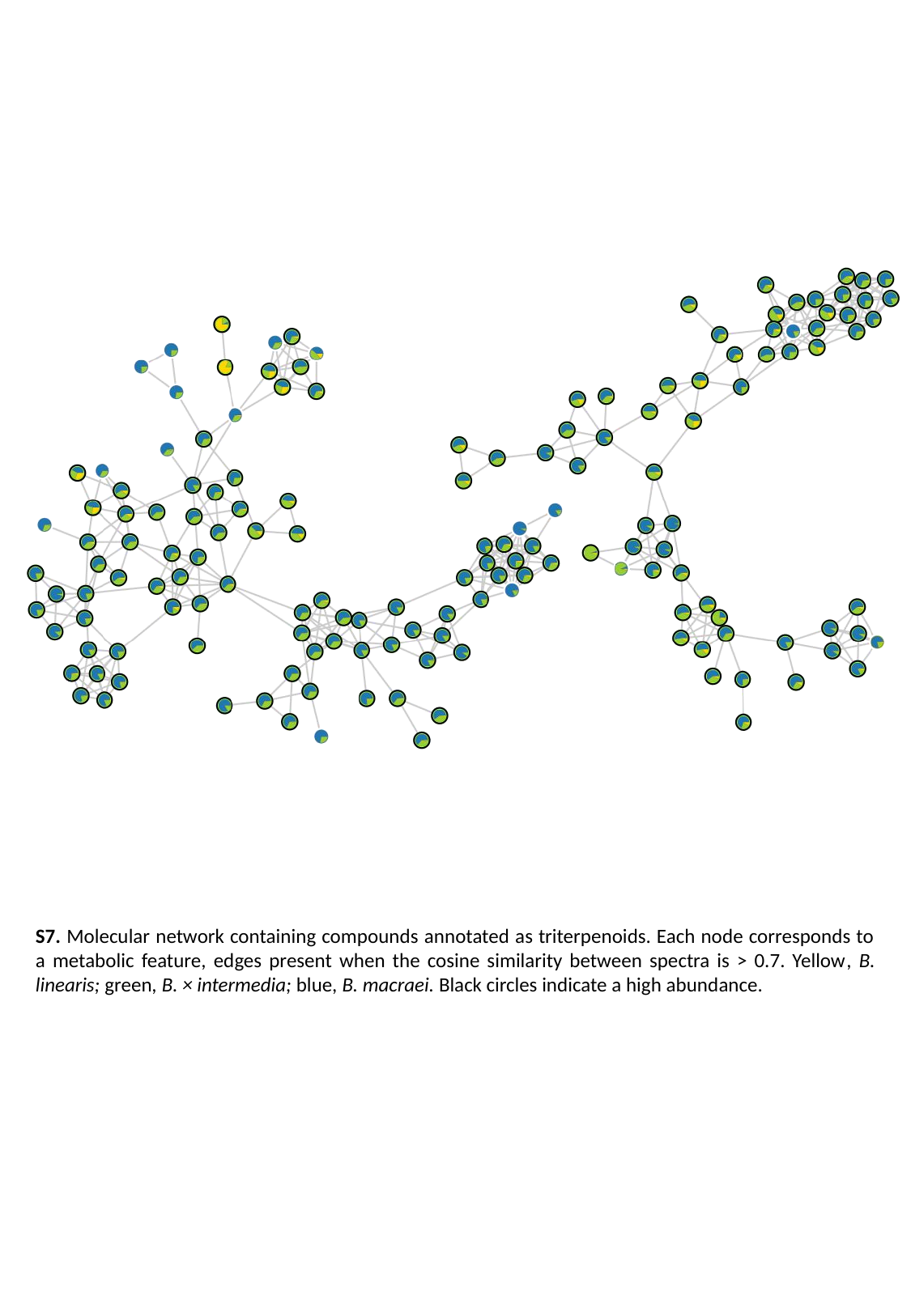

S7. Molecular network containing compounds annotated as triterpenoids. Each node corresponds to a metabolic feature, edges present when the cosine similarity between spectra is > 0.7. Yellow, B. linearis; green, B. × intermedia; blue, B. macraei. Black circles indicate a high abundance.

## Slide 8
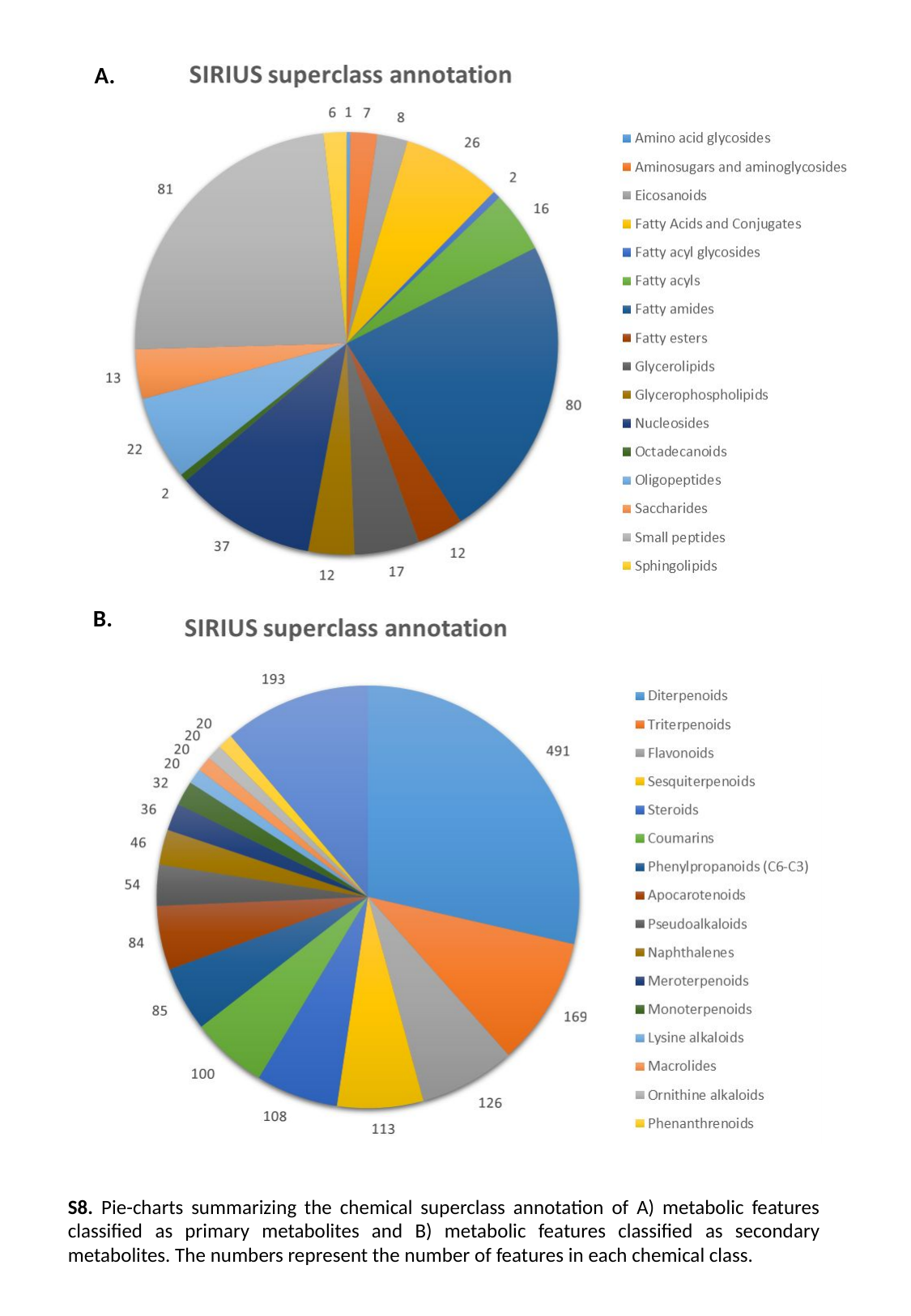

A.
B.
S8. Pie-charts summarizing the chemical superclass annotation of A) metabolic features classified as primary metabolites and B) metabolic features classified as secondary metabolites. The numbers represent the number of features in each chemical class.

## Slide 9
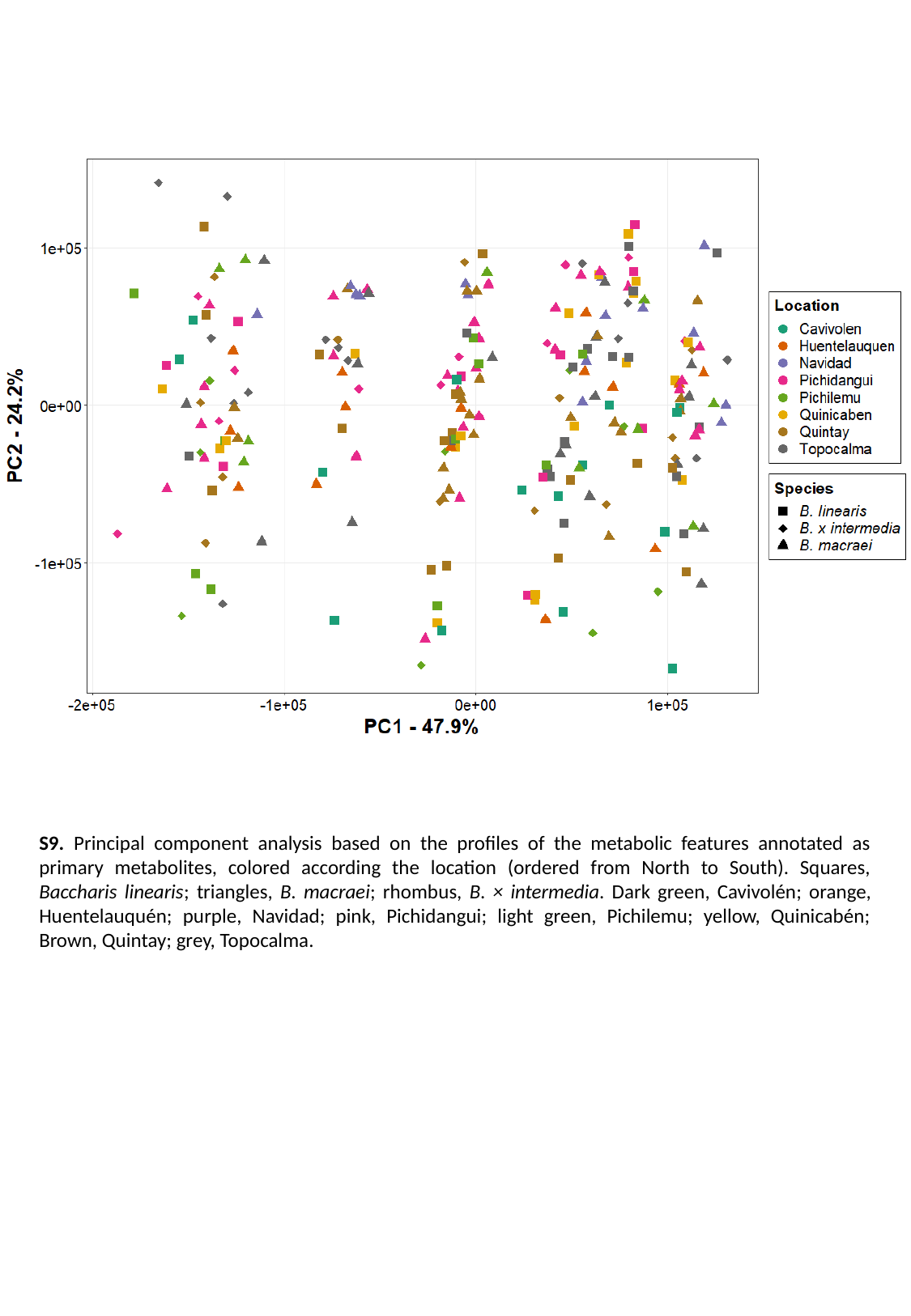

S9. Principal component analysis based on the profiles of the metabolic features annotated as primary metabolites, colored according the location (ordered from North to South). Squares, Baccharis linearis; triangles, B. macraei; rhombus, B. × intermedia. Dark green, Cavivolén; orange, Huentelauquén; purple, Navidad; pink, Pichidangui; light green, Pichilemu; yellow, Quinicabén; Brown, Quintay; grey, Topocalma.

## Slide 10
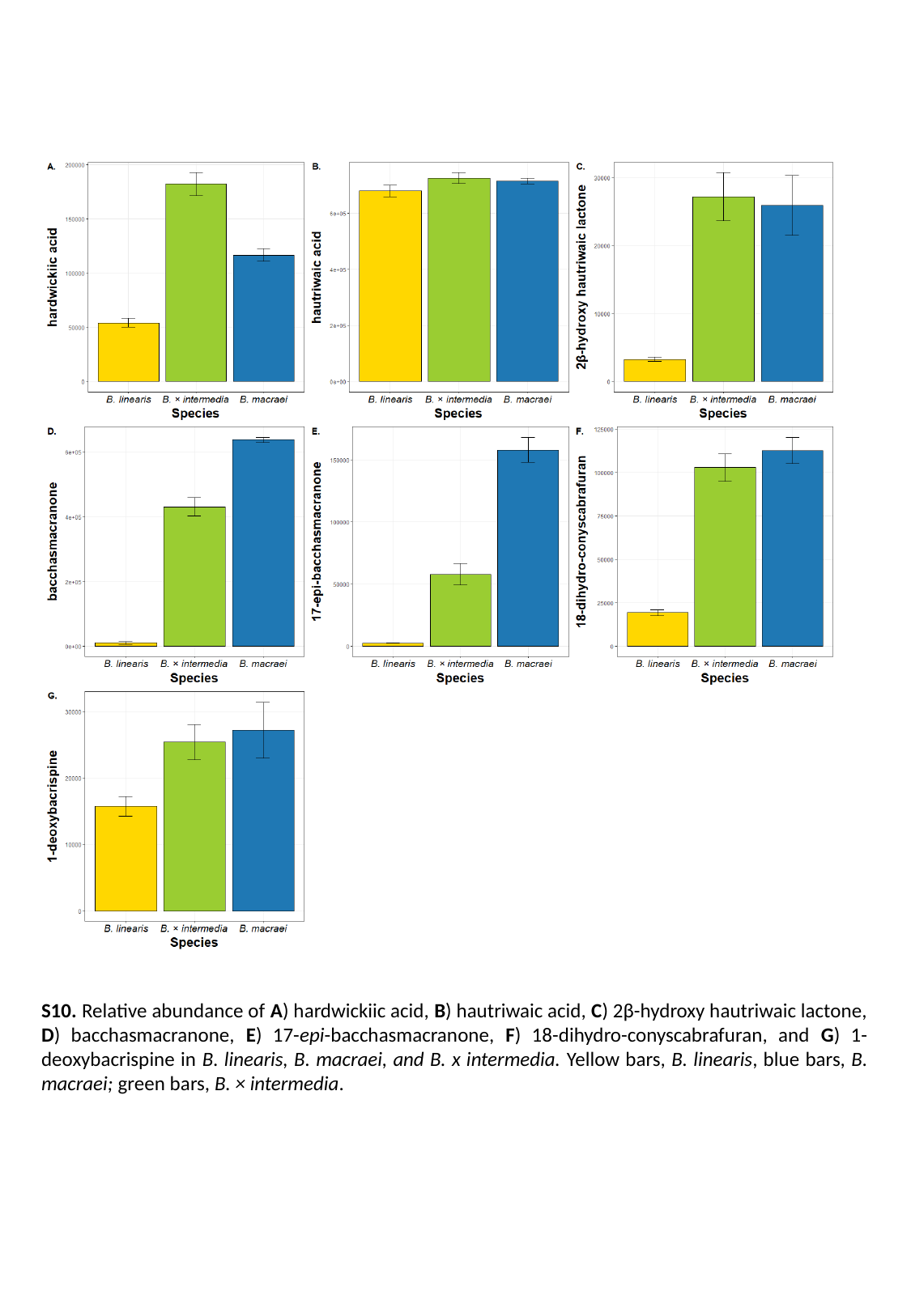

S10. Relative abundance of A) hardwickiic acid, B) hautriwaic acid, C) 2β-hydroxy hautriwaic lactone, D) bacchasmacranone, E) 17-epi-bacchasmacranone, F) 18-dihydro-conyscabrafuran, and G) 1-deoxybacrispine in B. linearis, B. macraei, and B. x intermedia. Yellow bars, B. linearis, blue bars, B. macraei; green bars, B. × intermedia.

## Slide 11
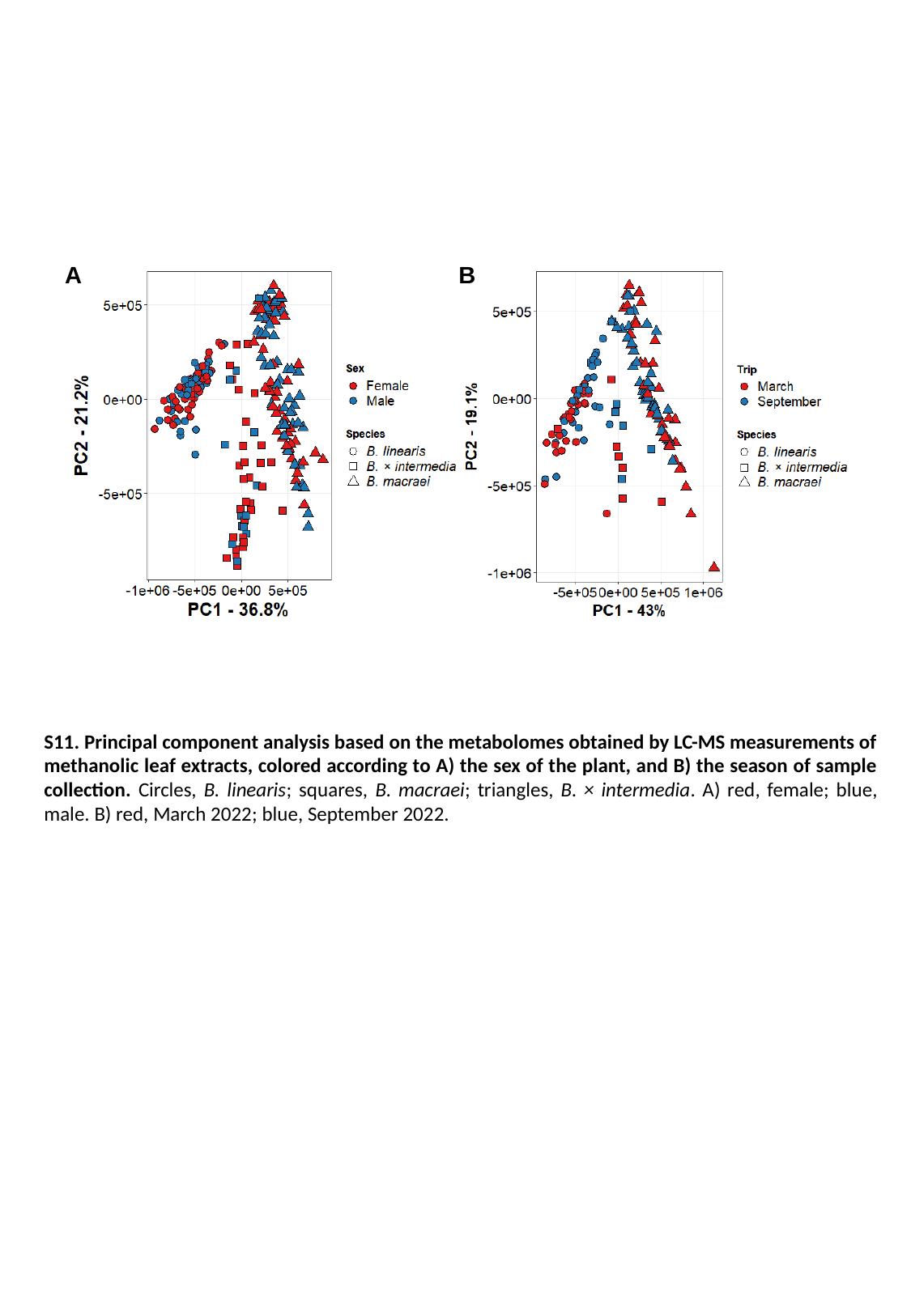

A
B
S11. Principal component analysis based on the metabolomes obtained by LC-MS measurements of methanolic leaf extracts, colored according to A) the sex of the plant, and B) the season of sample collection. Circles, B. linearis; squares, B. macraei; triangles, B. × intermedia. A) red, female; blue, male. B) red, March 2022; blue, September 2022.
